# Supplementary material for: Development of Novel Small-Molecule Activators of Pyruvate Kinase Muscle Isozyme 2, PKM2, to Reduce Photoreceptor Apoptosis
Source: Pharmaceuticals (Basel). 2023 May 6;16(5):705. doi: 10.3390/ph16050705 (PMC10222839; doi:10.3390/ph16050705)

File S1. Experimental Detail and NMR Spectra of PKM2 Activators

All purchased reagents were used without further purification. ^{1}H NMR spectra were recorded on a Bruker Advance 400 MHz NMR in deuterated dimethylsulfoxide. All compounds were either purified on a Teledyne ICSO CombiFlash system or by C18 reverse phase preparative HPLC column with solvent A (0.1% trifluoroacetic acid (TFA) in H2O) and solvent B (0.1% TFA in acetonitrile) as eluents.

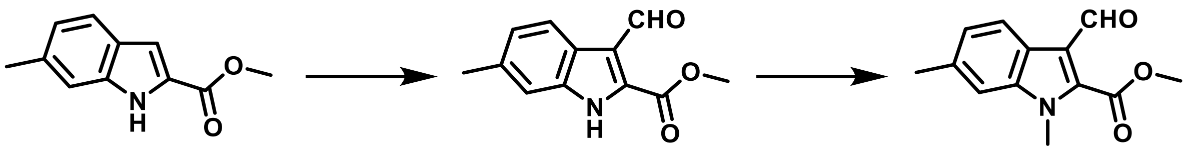

6 **8i** **8**

General Procedure A.

Intermediate 8i: Methyl 3-formyl-6-methyl-1H-indole-2-carboxylate.

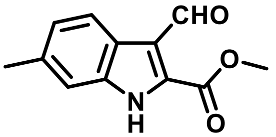

Phosphorous oxychloride (0.30 mL, 3.18 mmol) was added dropwise to DMF (6 mL) at 0 °C. The resulting reaction mixture was allowed to warm to room temperature and stirred for 10 minutes. The reaction mixture was cooled to 0 °C and methyl 6-methyl-1h-indole-2-carboxylate (**6**) (503 mg, 2.66 mmol) in DMF (3 mL) was added dropwise. The reaction mixture was heated to 80 °C and stirred for 1.5 hours, cooled to room temperature, and poured into 50 mL of ice water. The resulting precipitate was collected via vacuum filtration to provide the desired product (478 mg, 83%). ^{1}H NMR (400 MHz, DMSO-d6) δ 12.76 (s, 1H), 10.59 (s, 1H), 8.12 (d, J = 8.3 Hz, 1H), 7.37 – 7.34 (m, 1H), 7.15 (dd, J = 8.3, 1.4 Hz, 1H), 3.99 (s, 3H), 2.44 (s, 3H).

General Procedure B.

Intermediate 8: Methyl 3-formyl-1,6-dimethyl-1H-indole-2-carboxylate.

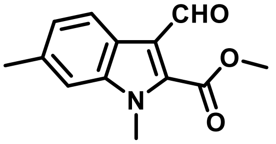

To a solution of methyl 3-formyl-6-methyl-1H-indole-2-carboxylate (**8i**) (478 mg, 2.20 mmol) in DMF (4 mL) was added K_2CO_3 (911 mg, 6.60 mmol) and iodomethane (0.15 mL, 2.42 mmol). The reaction mixture was stirred at room temperature overnight and poured into water (40 mL). The resulting precipitate was collected via vacuum filtration providing the desired product (451 mg, 87%). ^{1}H NMR (400 MHz, DMSO-d6) δ 10.41 (s, 1H), 8.18 (d, J = 8.2 Hz, 1H), 7.58 – 7.52 (m, 1H), 7.26 – 7.17 (m, 1H), 4.02 (s, 3H), 4.00 (s, 3H), 2.49 (s, 3H).

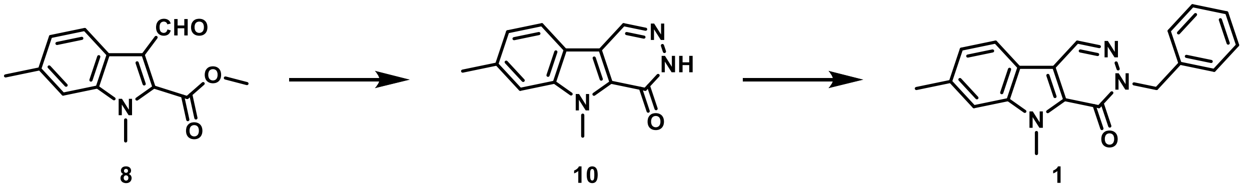

General Procedure C.

Intermediate 10: 5,7-Dimethyl-3,5-dihydro-4H-pyridazino[4,5-b]indol-4-one.

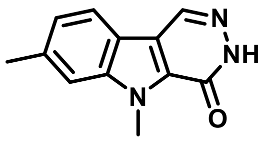

To a solution of methyl 3-formyl-1,6-dimethyl-1H-indole-2-carboxylate (**8**) (451 mg, 1.95 mmol) in ethoxyethanol (5 mL) was added hydrazine (7.81 mmol) and the reaction mixture was heated to 135 °C overnight. The reaction mixture was cooled to room temperature and water was added. The reaction mixture was extracted with ethyl acetate (EtOAc) (3 x 10 mL). The organics were combined, dried, concentrated and purified by flash column chromatography (0-20% MeOH in DCM) to provide the desired product (239 mg, 58%). ^{1}H NMR (400 MHz, DMSO-d6) δ 12.75 (s, 1H), 8.71 (s, 1H), 8.07 (d, J = 8.1 Hz, 1H), 7.58 – 7.52 (m, 1H), 7.23 (dd, J = 8.1, 1.4 Hz, 1H), 4.25 (s, 3H), 2.54 (s, 3H).

General Procedure D.

Compound 1: 3-Benzyl-5,7-dimethyl-3,5-dihydro-4H-pyridazino[4,5-b]indol-4-one.

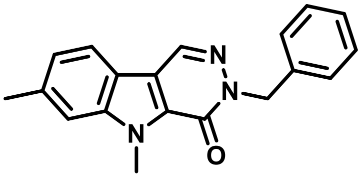

To a solution of 5,7-dimethyl-3,5-dihydro-4H-pyridazino[4,5-b]indol-4-one (**10**) (53 mg, 0.25 mmol) and KOtBu (35 mg, 0.29 mmol) in DMF (5 mL) at 0 °C was added benzyl bromide (51 mg, 0.30 mmol). The reaction mixture was warmed to room temperature and stirred overnight. Water was added and the reaction mixture was extracted with dichloromethane (3 x 10 mL). The organics were combined, dried, concentrated and purified by flash column chromatography (0-100% ethyl acetate (EtOAc) in hexanes) to provide the desired product (45 mg, 61 %). ^{1}H NMR (400 MHz, DMSO-d6) δ 8.78 (s, 1H), 8.08 (d, J = 8.0 Hz, 1H), 7.57 (s, 1H), 7.36 – 7.31 (m, 4H), 7.31 – 7.22 (m, 2H), 5.41 (s, 2H), 4.26 (s, 3H), 2.54 (s, 3H).

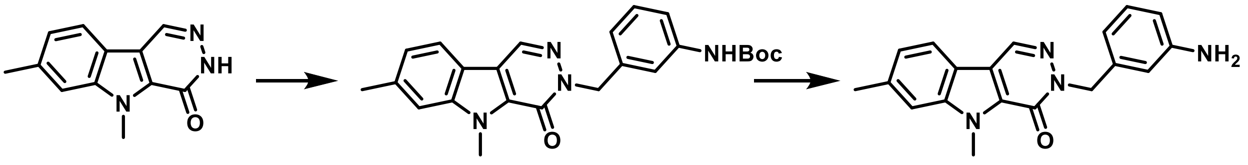

10 12 5

Intermediate 12: tert-butyl (3-((5,7-Dimethyl-4-oxo-4,5-dihydro-3H-pyridazino[4,5-b]indol-3-yl)methyl)phenyl)carbamate.

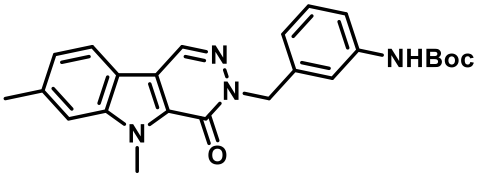

Intermediate 12 was prepared using general procedure D with tert-Butyl 3-(bromomethyl)phenylcarbamate to provide the desired product in 66% yield. ^{1}H NMR (400 MHz, DMSO-d6) δ 9.31 (s, 1H), 8.79 (s, 1H), 8.09 (d, J = 8.2 Hz, 1H), 7.57 (s, 1H), 7.44 – 7.38 (m, 1H), 7.39 – 7.31 (m, 1H), 7.26 – 7.22 (m, 1H), 7.22 – 7.16 (m, 1H), 6.95 – 6.89 (m, 1H), 5.34 (s, 2H), 4.26 (s, 3H), 2.54 (s, 3H), 1.44 (s, 9H).

General Procedure E.

Compound 5: 3-(3-Aminobenzyl)-5,7-dimethyl-3,5-dihydro-4H-pyridazino[4,5-b]indol-4-one.

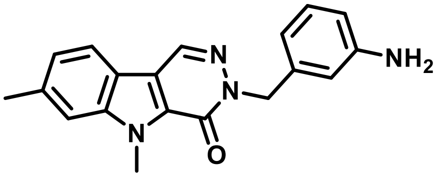

To a solution of tert-butyl (3-((5,7-Dimethyl-4-oxo-4,5-dihydro-3H-pyridazino[4,5-b]indol-3-yl)methyl)phenyl)carbamate (**12**) (53 mg, 0.13 mmol) in dichloromethane (5 mL) was added HCl (1 mL, 4 M HCl in dioxane) and the reaction mixture was stirred at room temperature for 1 hour. The reaction mixture was diluted with dichloromethane (10 mL), washed with saturated aqueous sodium bicarbonate (2 x 5 mL), dried, concentrated and purified by HPLC to provide the desired product (24 mg, 60 %). 1H NMR (400 MHz, DMSO-d6) δ 8.76 (s, 1H), 8.08 (dd, J = 8.1, 1.9 Hz, 1H), 7.56 (s, 1H), 7.27 – 7.21 (m, 1H), 7.00 – 6.91 (m, 1H), 6.49 – 6.41 (m, 3H), 5.24 (s, 2H), 5.05 (s, 1H), 4.25 (s, 3H), 2.54 (s, 3H).

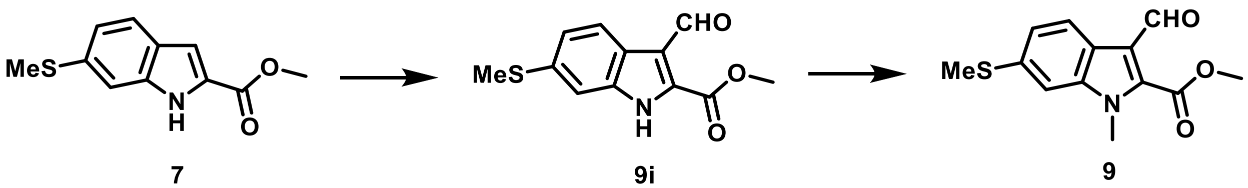

7 9i 9

Intermediate 9i: Methyl 3-formyl-6-(methylthio)-1H-indole-2-carboxylate.

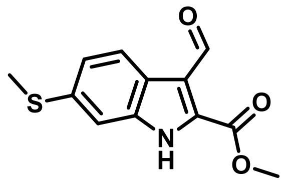

Intermediate 9i was generated via general procedure A in 83% yield. ^{1}H NMR (400 MHz, DMSO-d6) δ 12.79 (s, 1H), 10.58 (s, 1H), 8.15 (d, J = 8.5 Hz, 1H), 7.36 (d, J = 1.6 Hz, 1H), 7.23 (dd, J = 8.6, 1.7 Hz, 1H), 3.99 (s, 3H), 2.54 (s, 3H).

Intermediate 9: Methyl 3-formyl-1-methyl-6-(methylthio)-1H-indole-2-carboxylate.

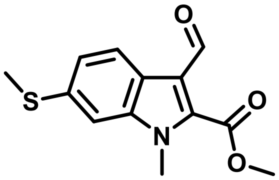

Intermediate 9 was generated via general procedure B in 63% yield. ^{1}H NMR (400 MHz, DMSO-d6) δ 10.41 (s, 1H), 8.19 (dd, J = 8.5, 0.6 Hz, 1H), 7.57 (d, J = 1.5 Hz, 1H), 7.27 (dd, J = 8.5, 1.6 Hz, 1H), 4.05 (s, 3H), 4.00 (s, 3H), 2.60 (s, 3H).

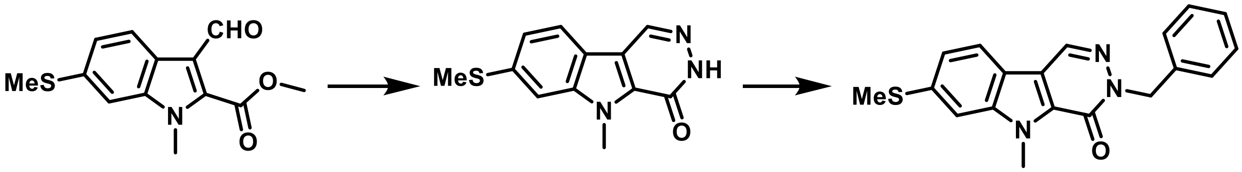

9 11 13

Intermediate 11: 5-Methyl-7-(methylthio)-3,5-dihydro-4H-pyridazino[4,5-b]indol-4-one.

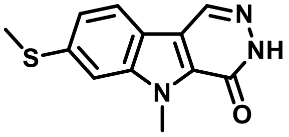

Intermediate 11 was prepared via general procedure C in 63% yield. ^{1}H NMR (400 MHz, DMSO-d6) δ 12.90 – 12.71 (m, 1H), 8.72 (s, 1H), 8.11 (dd, J = 8.4, 0.6 Hz, 1H), 7.57 (dd, J = 1.7, 0.7 Hz, 1H), 7.29 (dd, J = 8.4, 1.6 Hz, 1H), 4.27 (s, 3H), 2.63 (s, 3H).

Intermediate 13: 3-Benzyl-5-methyl-7-(methylthio)-3,5-dihydro-4H-pyridazino[4,5-b]indol-4-one.

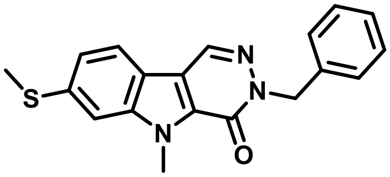

Intermediate 13 was prepared via general procedure D in 72% yield. ^{1}H NMR (400 MHz, DMSO-d6) δ 8.79 (s, 1H), 8.11 (dd, J = 8.4, 0.6 Hz, 1H), 7.57 (dd, J = 1.6, 0.6 Hz, 1H), 7.36 – 7.25 (m, 6H), 5.41 (s, 2H), 4.28 (s, 3H), 2.63 (s, 3H).

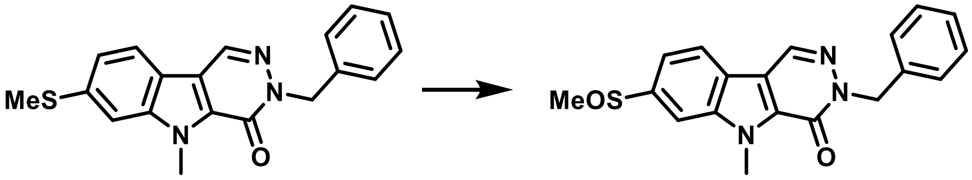

13 3

General Procedure F

Compound 3: 3-Benzyl-5-methyl-7-(methylsulfinyl)-3,5-dihydro-4H-pyridazino[4,5-b]indol-4-one.

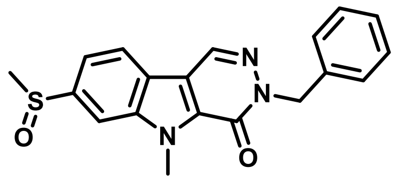

To a solution of 3-Benzyl-5-methyl-7-(methylthio)-3,5-dihydro-4H-pyridazino[4,5-b]indol-4-one (**13**) (27.0 mg, 0.081 mmol) in dichloromethane (10 mL) was added 3-chloroperoxybenzoic acid (13.9 mg, 0.081 mmol). The reaction mixture was stirred at room temperature overnight. The reaction mixture was diluted with dichloromethane (20 mL), extracted with saturated aqueous sodium bicarbonate (3 x 10 mL), dried, concentrated and purified by flash column chromatography (0-100 % EtOAc in hexanes) to provide the desired product (10 mg, 53%). ^{1}H NMR (400 MHz, DMSO-d6) δ 8.89 (s, 1H), 8.41 (dd, J = 8.4, 0.7 Hz, 1H), 8.11 (dd, J = 1.4, 0.7 Hz, 1H), 7.67 (dd, J = 8.3, 1.4 Hz, 1H), 7.37 – 7.31 (m, 4H), 7.31 – 7.25 (m, 1H), 5.43 (s, 2H), 4.35 (s, 3H), 2.85 (s, 3H).

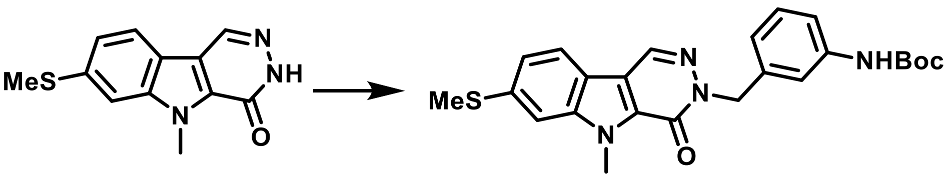

11 14

Intermediate 14: Tert-butyl (3-((5-methyl-7-(methylthio)-4-oxo-4,5-dihydro-3H-pyridazino[4,5-b]indol-3-yl)methyl)phenyl)carbamate.

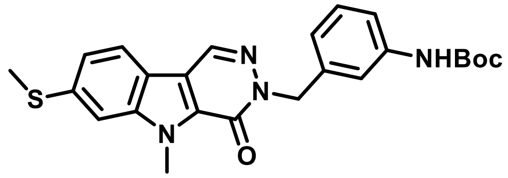

Intermediate 14 was prepared via general procedure D employing tert-Butyl 3-(bromomethyl)phenylcarbamate in 96% yield. The isolated material was ~30% impure but was advanced without additional purification. ^{1}H NMR (400 MHz, DMSO-d6) δ 9.31 (s, 1H), 8.79 (s, 1H), 8.15 – 8.10 (m, 1H), 7.59 – 7.54 (m, 1H), 7.44 – 7.39 (m, 1H), 7.33 – 7.26 (m, 1H), 7.22 – 7.16 (m, 1H), 7.17 – 7.07 (m, 1H), 6.94 – 6.89 (m, 1H), 5.34 (s, 2H), 4.27 (s, 3H), 2.63 (s, 3H), 1.45 (s, 9H).

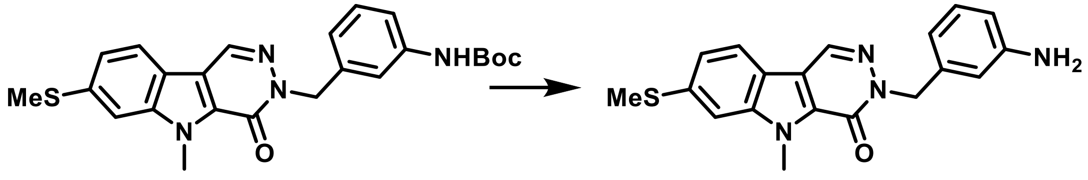

14 **4**

Compound 4: 3-Benzyl-5-methyl-7-(methylthio)-3,5-dihydro-4H-pyridazino[4,5-b]indol-4-one.

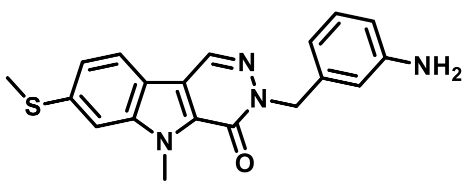

Compound 4 was prepared via general procedure E in 63% yield. ^{1}H NMR (400 MHz, DMSO-d6) δ 8.77 (s, 1H), 8.12 (dd, J = 8.4, 0.6 Hz, 1H), 7.57 (d, J = 1.6 Hz, 1H), 7.30 (dd, J = 8.4, 1.6 Hz, 1H), 7.03 – 6.90 (m, 1H), 6.54 – 6.39 (m, 3H), 5.24 (s, 2H), 5.05 (s, 2H), 4.28 (s, 3H), 2.63 (s, 3H).

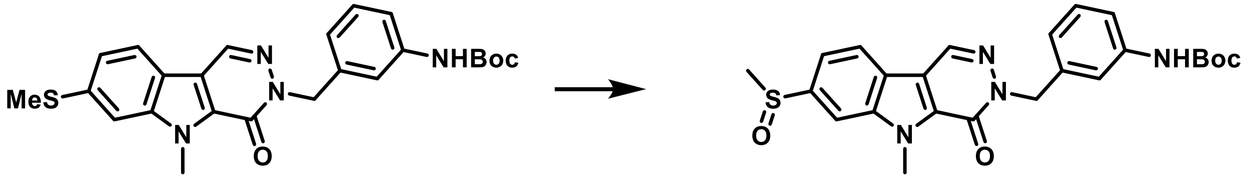

14 **15**

Intermediate 15: Tert-butyl (3-((5-methyl-7-(methylsulfinyl)-4-oxo-4,5-dihydro-3H-pyridazino[4,5-b]indol-3-yl)methyl)phenyl)carbamate.

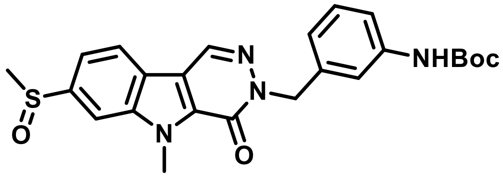

Intermediate 15 was generated via general procedure F in 57% yield. ^{1}H NMR (400 MHz, DMSO-d6) δ 9.31 (s, 1H), 8.90 (s, 1H), 8.45 – 8.39 (m, 1H), 8.14 – 8.08 (m, 1H), 7.71 – 7.64 (m, 1H), 7.44 – 7.39 (m, 1H), 7.39 – 7.33 (m, 1H), 7.23 – 7.17 (m, 1H), 6.96 – 6.89 (m, 1H), 5.36 (s, 2H), 4.34 (s, 3H), 2.86 (s, 3H), 1.45 (s, 9H).

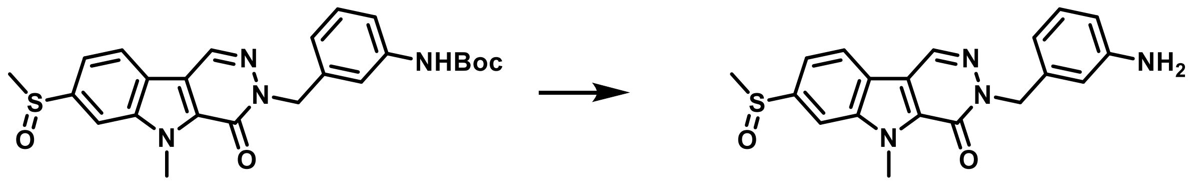

15 **2**

Compound 2: 3-(3-Aminobenzyl)-5-methyl-7-(methylsulfinyl)-3,5-dihydro-4H-pyridazino[4,5-b]indol-4-one.

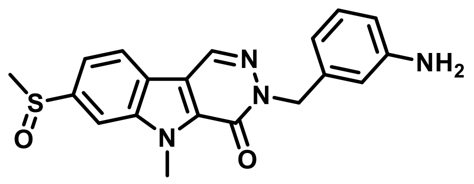

Compound 2 was generated via general procedure E in 75% yield. ^{1}H NMR (400 MHz, DMSO-d6) δ 8.91 (s, 1H), 8.42 (d, J = 8.3 Hz, 1H), 8.12 (d, J = 1.3 Hz, 1H), 7.73 – 7.64 (m, 1H), 7.28 – 7.20 (m, 1H), 7.03 – 6.94 (m, 1H), 6.93 – 6.83 (m, 2H), 5.38 (s, 2H), 4.35 (s, 3H), 2.85 (s, 3H).

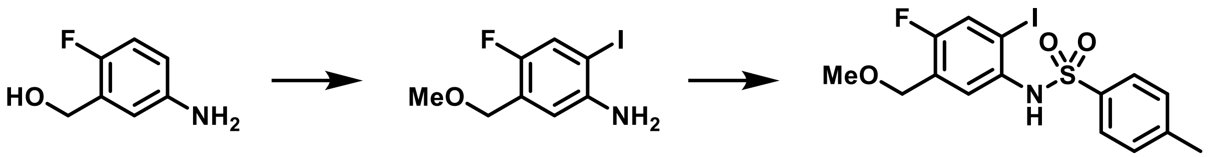

22 **23** **24**

Intermediate 23: 4-fluoro-2-iodo-5-(methoxymethyl)aniline

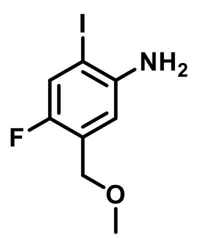

Intermediate 23 was generated by treating (5-amino-2-fluorophenyl)methanol (**22**) (1.68 g, 11.89 mmol) in THF (50 mL) with sodium hydride (342 mg, 14.27 mmol). The resulting reaction mixture was cooled to 0 °C and iodomethane (1.69 g, 11.89 mmol) was added. The reaction mixture was allowed to warm to room temperature and stirred for 2 hours. Saturated aqueous sodium chloride was added to the reaction mixture and extracted with ethyl acetate. The organics were combined, dried, and concentrated to provide 4-fluoro-3-(methoxymethyl)aniline, which was taken on without purification. 4-fluoro-3-(methoxymethyl)aniline (**23**) (1.84 g, 11.89 mmol) in acetic acid was treated with 1-iodopyrrolidine-2,5-dione (3.08 g, 13.78 mmol) and the reaction mixture was stirred at room temperature for 3 hours. The reaction mixture was diluted with EtOAc, washed with saturated aqueous sodium thiosulfate and purified by flash column chromatography (0-50% EtOAc in hexanes) to provide the desired product in 68% yield. 1H NMR (400 MHz, DMSO-d6) δ 7.39 (d, J = 9.3 Hz, 1H), 6.83 – 6.75 (m, 1H), 5.08 (s, 2H), 4.36 – 4.26 (m, 2H), 3.29 (s, 3H).

Intermediate 24: N-(4-fluoro-2-iodo-5-(methoxymethyl)phenyl)-4-methylbenzenesulfonamide

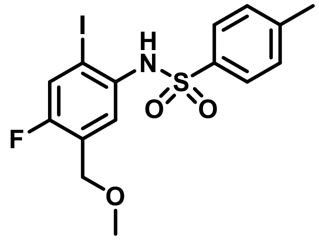

Intermediate 24 was generated by treating intermediate 23 (2.30 g, 8.18 mmol) in dichloromethane (50 mL) with pyridine (1.00 mL, 12.27 mmol) and 4-methylbenzenesulfonyl chloride (1.56 g, 8.18 mmol), and the reaction mixture stirred at room temperature overnight. The reaction mixture was washed with saturated aqueous sodium bicarbonate, dried, concentrated and purified by FCC (0-100% EtOAc in hexanes) to provide the desired product in 71% yield. 1H NMR (400 MHz, DMSO-d6) δ 9.69 (s, 1H), 7.73 – 7.66 (m, 1H),

7.62 – 7.53 (m, 2H), 7.43 – 7.34 (m, 2H), 7.02 – 6.96 (m, 1H), 4.36 – 4.27 (m, 2H), 3.19 (s, 3H), 2.38 (s, 3H).

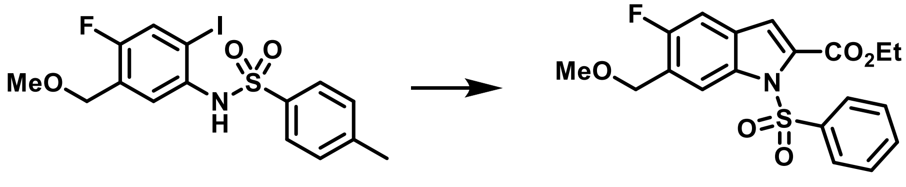

24 25

Intermediate 25: ethyl 5-fluoro-6-(methoxymethyl)-1-tosyl-1H-indole-2-carboxylate

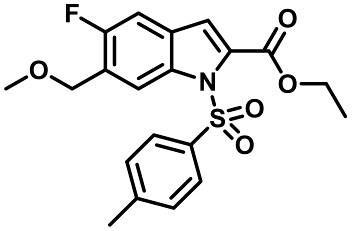

Intermediate 25 was generated by treating intermediate 24 (3.11 g, 7.15 mmol) in degassed THF (50 mL) with $Pd(PPh_3)_4$ (413 mg, 0.36 mmol), ethyl propiolate (2.10 g, 21.44 mol), DIPEA (4.62 g, 35.73 mmol) and $ZnBr_2$ (4.83 g, 21.44 mmol). The reaction mixture was heated to 80 °C overnight, cooled to room temperature and filtered through celite. The filtrate was washed with saturated sodium bicarbonate, dried, concentrated and purified by flash column chromatography (0-100% EtOAc in hexanes) to provide the desired product in 65% yield. 1H NMR (400 MHz, DMSO-d6) δ 8.07 – 8.02 (m, 1H), 7.85 – 7.81 (m, 2H), 7.51 (d, J = 9.8 Hz, 1H), 7.46 – 7.41 (m, 2H), 7.35 (d, J = 0.8 Hz, 1H), 4.60 (d, J = 1.2 Hz, 2H), 4.36 (q, J = 7.1 Hz, 2H), 3.35 (s, 3H), 2.36 (s, 3H), 1.32 (t, J = 7.1 Hz, 3H).

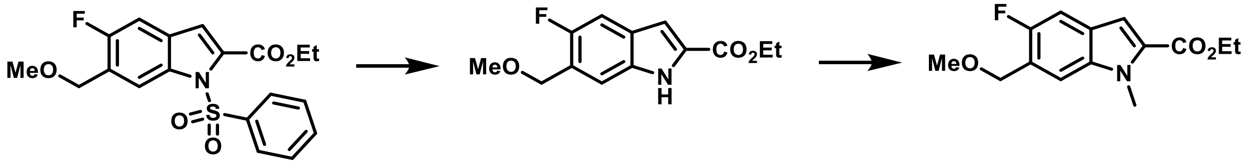

25 26i 26

Intermediate 26i: ethyl 5-fluoro-6-(methoxymethyl)-1H-indole-2-carboxylate

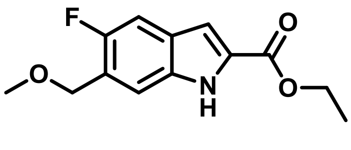

Intermediate 26i was generated by treating intermediate 25 (2.30 g, 5.68 mmol) in ethanol (25 mL) and THF (25 mL) with potassium hydroxide (796 mg, 14.19 mmol). The reaction mixture was stirred at room temperature for 2 hours. The reaction mixture was diluted with EtOAc and washed with water, dried, concentrated and purified by flash column chromatography (0-10% MeOH in DCM) to provide the desired product in 78% yield. 1H NMR (400 MHz, DMSO-d6) δ 12.00 (s, 1H), 7.47 (dd, J = 6.2, 0.9 Hz, 1H), 7.43 (d, J = 10.8 Hz, 1H), 7.12 (dd, J = 2.2, 0.9 Hz, 1H), 4.58 – 4.51 (m, 2H), 4.35 (q, J = 7.1 Hz, 2H), 3.34 (s, 3H), 1.35 (t, J = 7.1 Hz, 3H).

Intermediate 26: ethyl 5-fluoro-6-(methoxymethyl)-1-methyl-1H-indole-2-carboxylate

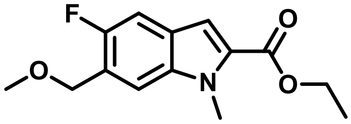

Intermediate 26 was generated by treating intermediate 26i (50.4 mg, 0.20 mmol) in DMF (3 mL) with K_2CO_3 (69.3 mg, 0.50 mmol) and MeI (31.3 mg, 0.22 mmol). The resulting reaction mixture was stirred overnight at room temperature. The reaction mixture was diluted with EtOAc, washed with water, dried, concentrated and purified by flash column chromatography (0-50% EtOAc in hexanes) to provide the desired product in 92% yield. 1H NMR (400 MHz, DMSO-d6) δ 7.64 (d, J = 6.0 Hz, 1H), 7.47 (d, J = 10.5 Hz, 1H), 7.24 (d, J = 0.8 Hz, 1H), 4.57 (s, 2H), 4.33 (q, J = 7.1 Hz, 2H), 4.04 (s, 3H), 3.36 (s, 3H), 1.34 (t, J = 7.1 Hz, 3H).

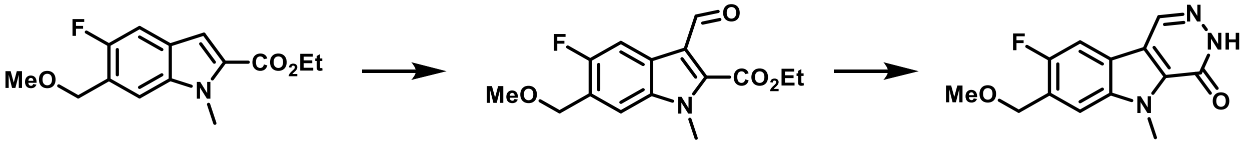

26 **27i** **27**

Intermediate 27i: ethyl 5-fluoro-3-formyl-6-(methoxymethyl)-1-methyl-1H-indole-2-carboxylate

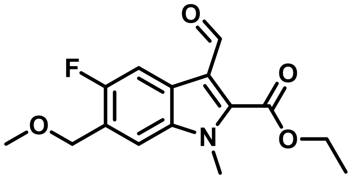

Intermediate 27i was generated from intermediate 26 via general procedure A in 73% yield. ^{1}H NMR (400 MHz, DMSO-d6) δ 10.42 (s, 1H), 7.96 (d, J = 10.5 Hz, 1H), 7.82 (d, J = 6.0 Hz, 1H), 4.60 (t, J = 0.9 Hz, 2H), 4.48 (qd, J = 7.1, 1.7 Hz, 2H), 4.07 (s, 3H), 3.38 (s, 3H), 1.41 (td, J = 7.1, 1.0 Hz, 3H).

Intermediate 27: 8-fluoro-7-(methoxymethyl)-5-methyl-3,5-dihydro-4H-pyridazino[4,5-b]indol-4-one

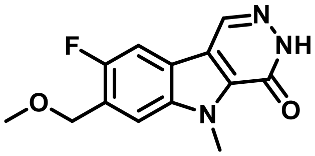

Intermediate 27 was generated from intermediate 27i via general procedure C in 89% yield. 1H NMR (400 MHz, DMSO-d6) δ 12.80 (s, 1H), 8.72 (s, 1H), 8.07 (d, J = 10.2 Hz, 1H), 7.80 (d, J = 5.9 Hz, 1H), 4.65 (d, J = 1.2 Hz, 2H), 4.29 (s, 3H), 3.40 (s, 3H).

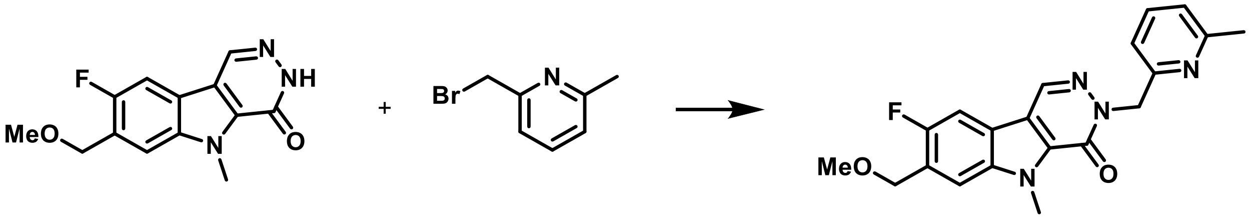

27 28 29

Intermediate 29: 8-fluoro-7-(methoxymethyl)-5-methyl-3-((6-methylpyridin-2-yl)methyl)-3,5-dihydro-4H-pyridazino[4,5-b]indol-4-one

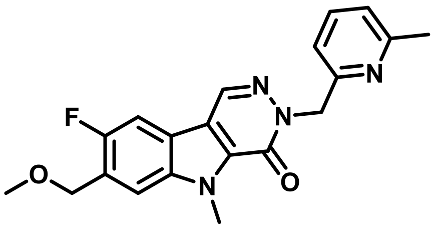

Intermediate 29 was generated by treating intermediate 27 (25.2 mg, 0.097 mmol) in DMF (3 mL) with 2-(bromomethyl)-6-methyl-pyridine (**28**) (19.7 mg, 0.106 mmol) and Cs_2CO_3 (78.6 mg, 0.241 mmol). The reaction mixture was stirred overnight at room temperature, diluted with EtOAc, washed with water, dried, concentrated and purified by flash column chromatography (0-100% EtOAc in hexanes) to provide the desired production 77% yield. 1H NMR (400 MHz, DMSO-d6) δ 8.82 (s, 1H), 8.10 (d, J = 10.2 Hz, 1H), 7.83 (d, J = 5.8 Hz, 1H), 7.60 (t, J = 7.7 Hz, 1H), 7.14 (d, J = 7.6 Hz, 1H), 6.85 (d, J = 7.7 Hz, 1H), 5.46 (s, 2H), 4.66 (d, J = 1.0 Hz, 2H), 4.30 (s, 3H), 3.39 (d, J = 13.3 Hz, 3H), 2.45 (s, 3H).

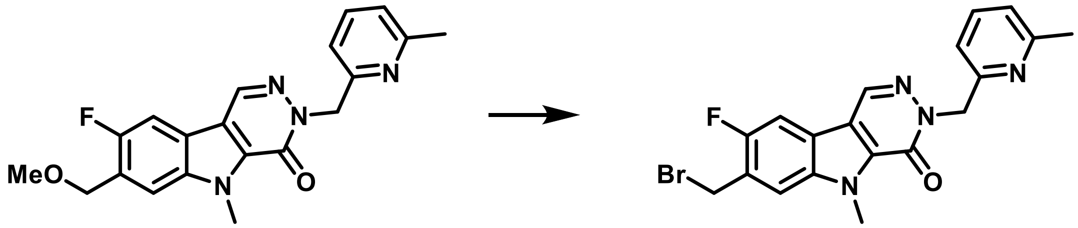

29 30

General procedure G

Intermediate 30: 7-(bromomethyl)-8-fluoro-5-methyl-3-((6-methylpyridin-2-yl)methyl)-3,5-dihydro-4H-pyridazino[4,5-b]indol-4-one

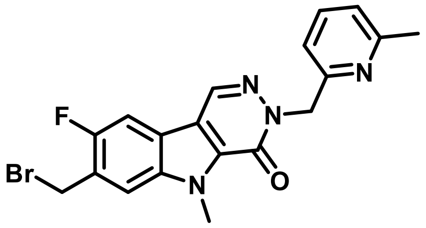

Intermediate 30 was generated by treating intermediate 29 (150 mg, 0.41 mmol) in dichloromethane (5 mL) with boron tribromide (461 mg, 1.843 mmol) and the reaction mixture was stirred for 2 hours at room temperature. The reaction mixture was diluted with dichloromethane, washed with saturated aqueous sodium bicarbonate, dried, concentrated and used without further purification. 1H NMR (400 MHz, DMSO-d6) δ 8.82 (s, 1H), 8.15 (d, J = 10.2 Hz, 1H), 8.04 (d, J = 6.1 Hz, 1H), 7.61 (t, J = 7.7 Hz, 1H), 7.15 (d, J = 7.7 Hz, 1H), 6.86 (d, J = 7.8 Hz, 1H), 5.46 (s, 2H), 4.92 (d, J = 1.2 Hz, 2H), 4.28 (s, 3H), 2.44 (s, 3H).

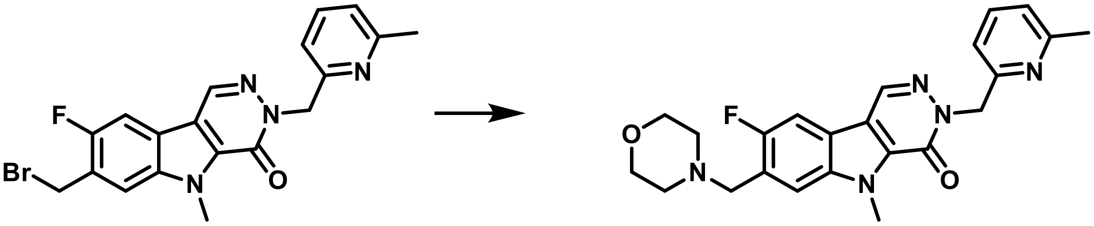

30 **16**

General procedure H

Compound 16: 8-fluoro-5-methyl-3-((6-methylpyridin-2-yl)methyl)-7-(morpholinomethyl)-3,5-dihydro-4H-pyridazino[4,5-b]indol-4-one

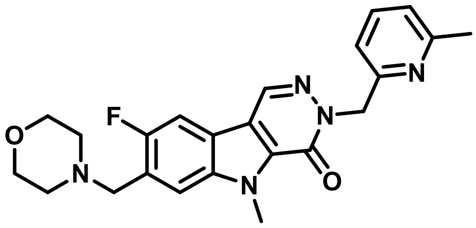

Compound 16 was generated by treating intermediate 30 (38.1 mg, 0.068 mmol) in dichloromethane (3 mL) with triethylamine (27.4 mg, 0.271 mmol) and morpholine (11.8 mg, 0.135 mmol) and the reaction mixture was stirred at room temperature overnight. The reaction mixture was diluted with dichloromethane, washed with saturated aqueous sodium bicarbonate, dried, concentrated and purified by flash column chromatography (0-20% MeOH in DCM) to provide the desired product 90 % over two steps. 1H NMR (400 MHz, DMSO-d6) δ 8.81 (s, 1H), 8.08 (d, J = 10.1 Hz, 1H), 7.78 (d, J = 5.9 Hz, 1H), 7.60 (t, J = 7.7 Hz, 1H), 7.14 (d, J = 7.6 Hz, 1H), 6.84 (d, J = 7.7 Hz, 1H), 4.29 (s, 3H), 3.75 – 3.69 (m, 2H), 3.65 – 3.57 (m, 6H), 2.47 (t, J = 4.6 Hz, 4H), 2.44 (s, 3H).

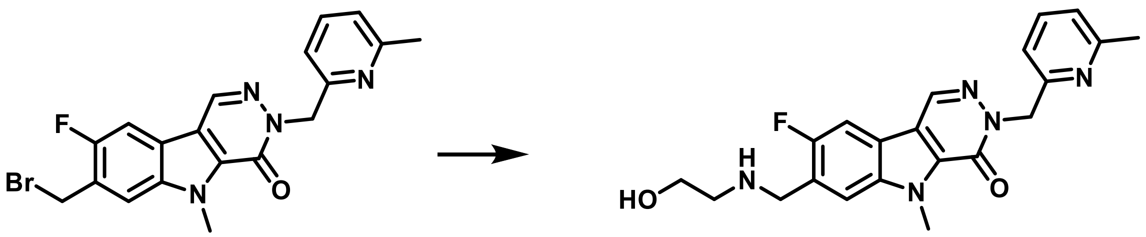

30 17

Compound 17: 8-fluoro-7-(((2-hydroxyethyl)amino)methyl)-5-methyl-3-((6-methylpyridin-2-yl)methyl)-3,5-dihydro-4H-pyridazino[4,5-b]indol-4-one

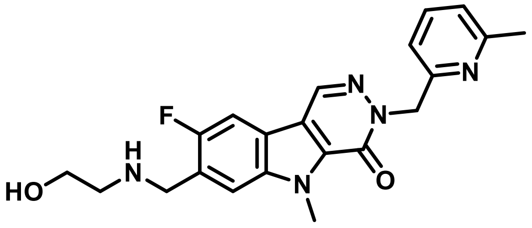

Compound 17 was generated using general procedure H and employing ethanolamine as the nucleophile in 62% yield over two steps. 1H NMR (400 MHz, DMSO-d6) δ 8.80 (s, 1H), 8.05 (d, J = 10.3 Hz, 1H), 7.85 (d, J = 6.0 Hz, 1H), 7.60 (t, J = 7.7 Hz, 1H), 7.14 (d, J = 7.6 Hz, 1H), 6.84 (d, J = 7.7 Hz, 1H), 5.46 (s, 2H), 4.52 (t, J = 5.4 Hz, 1H), 4.29 (s, 3H), 3.95 (s, 2H), 3.52 (q, J = 5.6 Hz, 2H), 2.67 (t, J = 5.7 Hz, 2H), 2.53 – 2.51 (m, 1H), 2.45 (s, 3H).

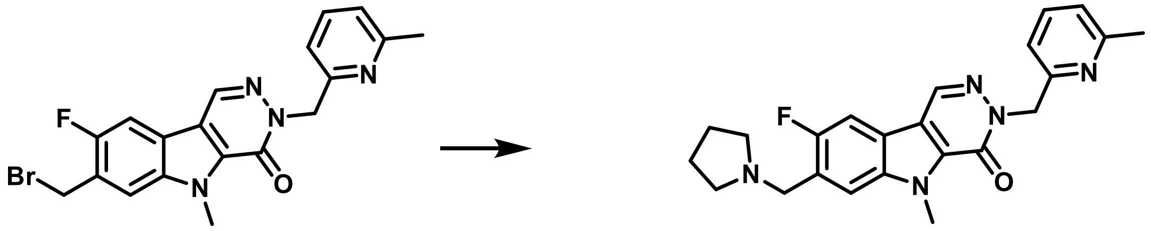

30 18

Compound 18: 8-fluoro-5-methyl-3-((6-methylpyridin-2-yl)methyl)-7-(12yrrolidine-1-ylmethyl)-3,5-dihydro-4H-pyridazino[4,5-b]indol-4-one

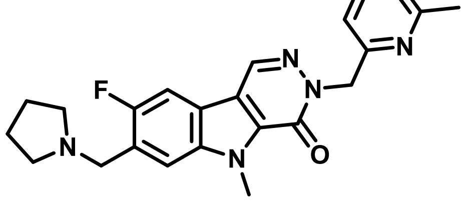

Compound 18 was generated from intermediate 30 via general procedure H and employing pyrrolidine as the nucleophile in 59% yield. 1H NMR (400 MHz, DMSO-d6) δ 8.80 (s, 1H), 8.06 (d, J = 10.2 Hz, 1H), 7.77 (d, J = 5.9 Hz, 1H), 7.60 (t, J = 7.7 Hz, 1H), 7.14 (d, J = 7.6 Hz, 1H), 6.84 (d, J = 7.7 Hz, 1H), 5.46 (s, 2H), 4.29 (s, 3H), 3.82 (s, 2H), 2.54 (s, 4H), 2.44 (s, 3H), 1.73 (s, 4H).

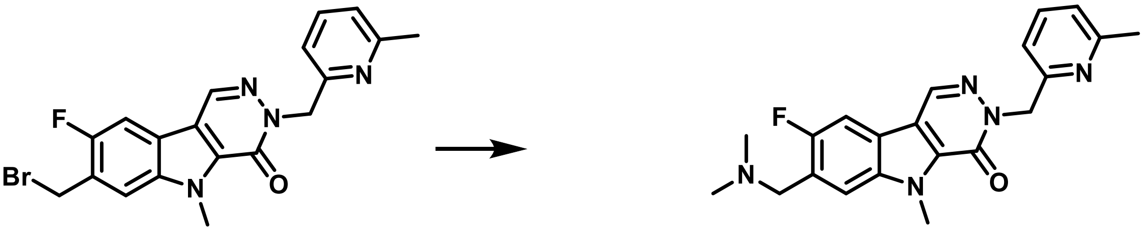

30 **19**

Compound 19: 7-((dimethylamino)methyl)-8-fluoro-5-methyl-3-((6-methylpyridin-2-yl)methyl)-3,5-dihydro-4H-pyridazino[4,5-b]indol-4-one

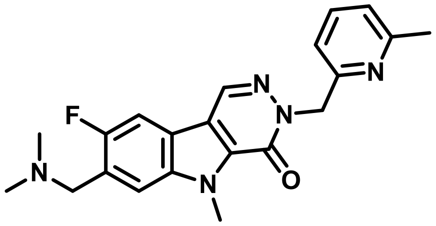

Compound 19 was generated from intermediate 30 via general procedure H and employing dimethylamine hydrochloride as the nucleophile in 78% yield. 1H NMR (400 MHz, DMSO-d6) δ 8.81 (s, 1H), 8.07 (d, J = 10.2 Hz, 1H), 7.77 (d, J = 5.9 Hz, 1H), 7.60 (t, J = 7.7 Hz, 1H), 7.14 (d, J = 7.7 Hz, 1H), 6.84 (d, J = 7.8 Hz, 1H), 5.46 (s, 2H), 4.29 (s, 3H), 3.68 – 3.60 (m, 2H), 2.44 (s, 3H), 2.23 (d, J = 1.5 Hz, 6H).

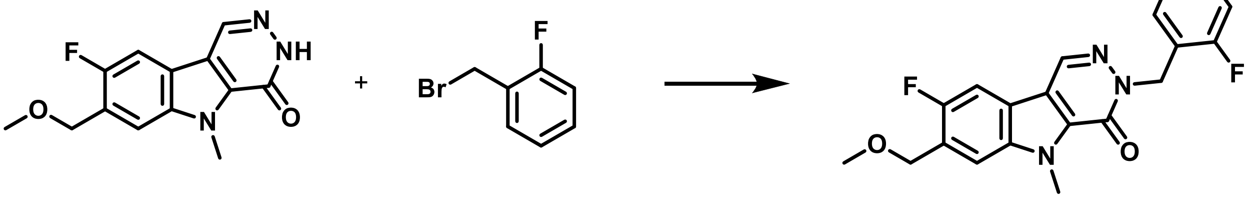

27 **31** **32**

Intermediate 32: 8-fluoro-3-(2-fluorobenzyl)-7-(methoxymethyl)-5-methyl-3,5-dihydro-4H-pyridazino[4,5-b]indol-4-one

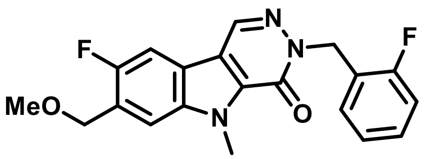

Intermediate 32 was generated from Intermediate 27 and 2-fluorobenzyl bromide (**31**) as the electrophile via general procedure D in 77% yield. 1H NMR (400 MHz, DMSO-d6) δ 8.80 (s, 1H), 8.08 (d, J = 10.2 Hz, 1H), 7.82 (d, J = 5.9 Hz, 1H), 7.34 (tdd, J = 7.4, 5.4, 2.0 Hz, 1H), 7.29 – 7.09 (m, 3H), 5.46 (s, 2H), 4.68 – 4.60 (m, 2H), 4.29 (s, 3H), 3.40 (s, 3H).

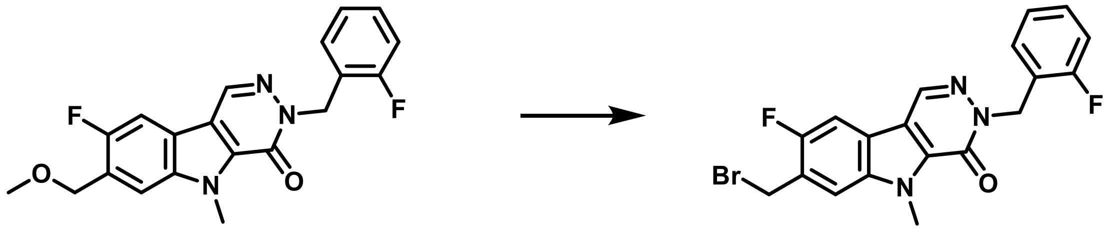

32 **33**

Intermediate 33: 7-(bromomethyl)-8-fluoro-3-(2-fluorobenzyl)-5-methyl-3,5-dihydro-4H-pyridazino[4,5-b]indol-4-one

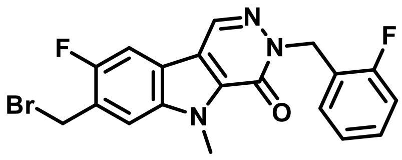

Intermediate 33 was generated from intermediate 32 via general procedure G. 1H NMR (400 MHz, DMSO-d6) δ 8.80 (s, 1H), 8.13 (d, J = 10.2 Hz, 1H), 8.03 (d, J = 6.2 Hz, 1H), 7.41 – 7.30 (m, 1H), 7.30 – 7.07 (m, 3H), 5.46 (s, 2H), 4.91 (d, J = 1.1 Hz, 2H), 4.28 (s, 3H).

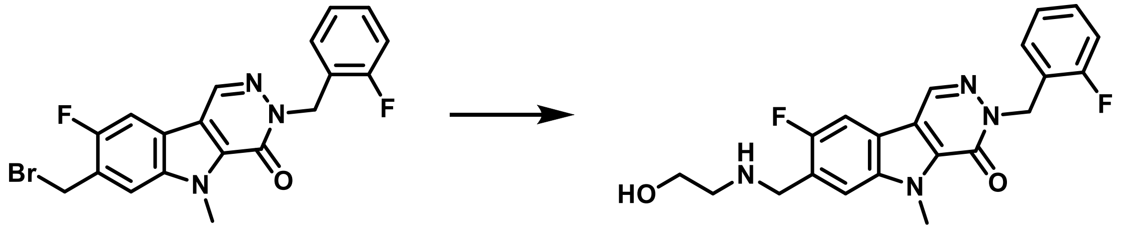

33 **20**

Compound 20: 8-fluoro-3-(2-fluorobenzyl)-7-(((2-hydroxyethyl)amino)methyl)-5-methyl-3,5-dihydro-4H-pyridazino[4,5-b]indol-4-one

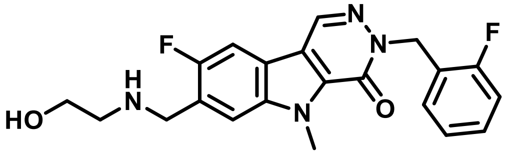

Compound 20 was generated from intermediate 33 via general procedure H and employing ethanolamine as the nucleophile in 65% yield over two steps. 1H NMR (400 MHz, DMSO-d6) δ 8.79 (s, 1H), 8.05 (d, J = 10.2 Hz, 1H), 7.88 (d, J = 6.0 Hz, 1H), 7.42 – 7.32 (m, 1H), 7.28 – 7.17 (m, 2H), 7.14 (td, J = 7.4, 1.2 Hz, 1H), 5.47 (s, 2H), 4.63 (s, 1H), 4.48-2.04 (bs, 1H), 4.29 (s, 3H), 4.01 (s, 2H), 3.54 (q, J = 5.5 Hz, 2H), 2.77 – 2.70 (m, 2H).

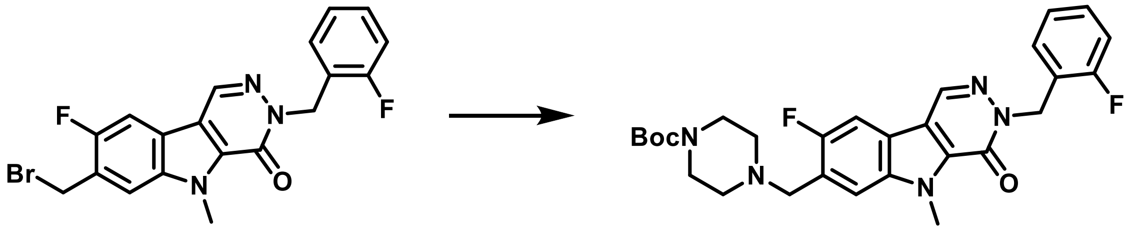

33 **34**

Intermediate 34: tert-butyl 4-((8-fluoro-3-(2-fluorobenzyl)-5-methyl-4-oxo-4,5-dihydro-3H-pyridazino[4,5-b]indol-7-yl)methyl)piperazine-1-carboxylate

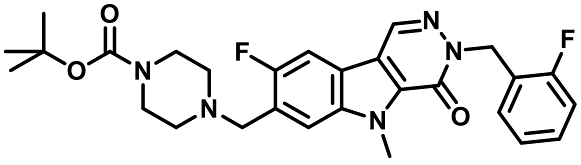

Intermediate 34 was generated from intermediate 33 via general procedure H and employing N-Boc-piperazine as the nucleophile in 56% yield. 1H NMR (400 MHz, DMSO-d6) δ 8.79 (s, 1H), 8.06 (d, J = 10.1 Hz, 1H), 7.77 (d, J = 5.8 Hz, 1H), 7.40 – 7.29 (m, 1H), 7.28 – 7.10 (m, 3H), 5.47 (s, 2H), 4.29 (s, 3H), 3.73 (s, 2H), 3.41 – 3.32 (m, 4H), 2.46 – 2.39 (m, 4H), 1.39 (s, 9H).

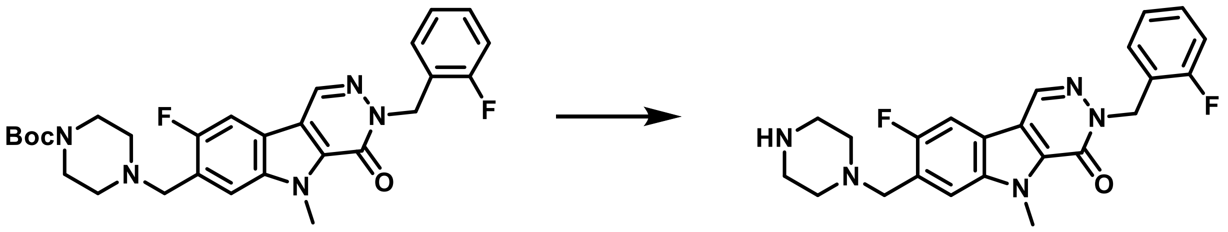

34 **21**

Compound 21: 8-fluoro-3-(2-fluorobenzyl)-5-methyl-7-(piperazin-1-ylmethyl)-3,5-dihydro-4H-pyridazino[4,5-b]indol-4-one

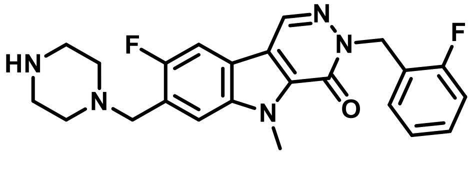

Compound 21 was generated from intermediate 34 via removal of the tert-butylcarbamate (boc) protecting group under acid mediated conditions as described in general procedure E in 92% yield. 1H NMR (400 MHz, DMSO-d6) δ 8.81 (s, 1H), 8.66 (s, 2H), 8.13 (d, J = 10.1 Hz, 1H), 7.87 (d, J = 5.9 Hz, 1H), 7.41 – 7.30 (m, 1H), 7.26 – 7.08 (m, 3H), 5.47 (s, 2H), 4.30 (s, 3H), 4.00 (s, 2H), 3.24 – 3.15 (m, 4H), 2.95 – 2.79 (m, 4H)

NMR Spectra

The NMR spectrum for all compounds were obtained from a Bruker® 400 MHz NMR in either deuterated dimethylsulfoxide or methanol.

Intermediate 8i

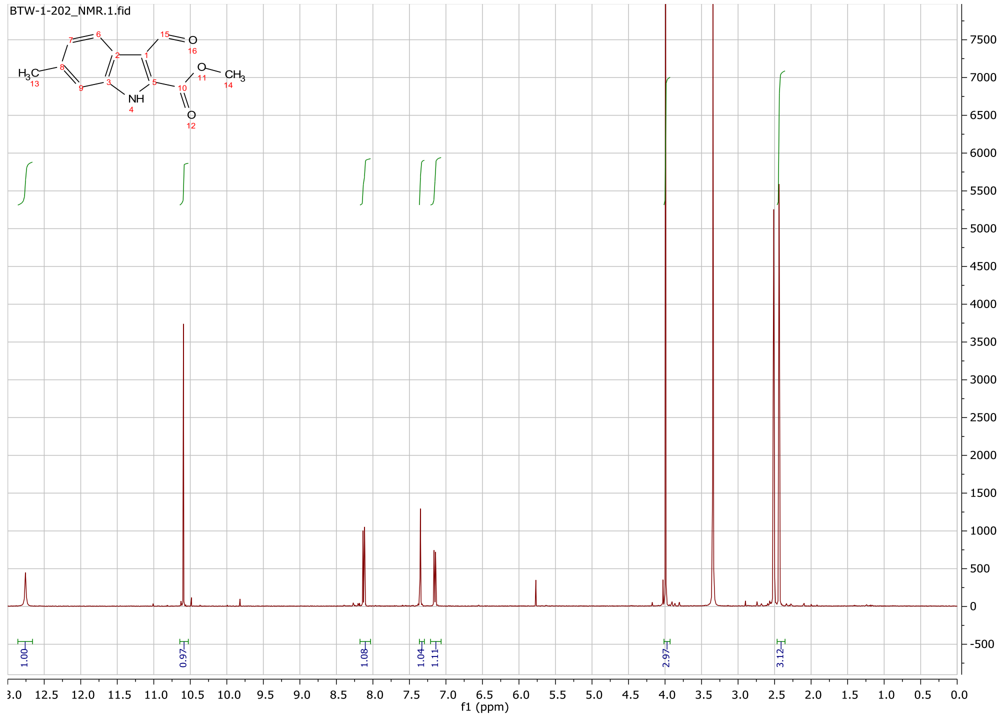

Intermediate 8

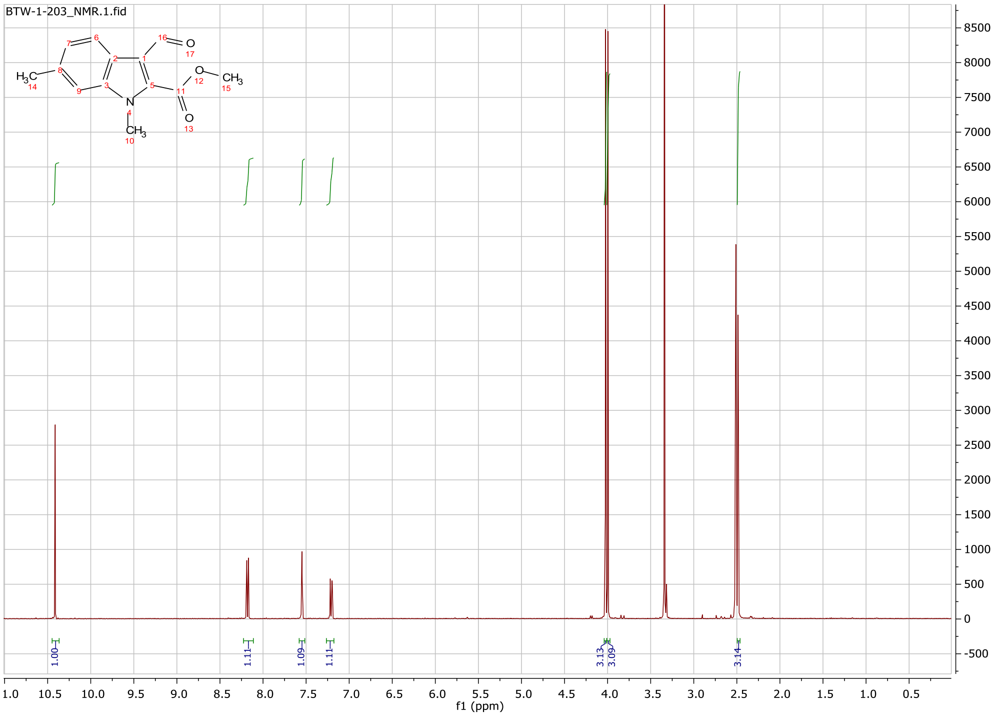

Intermediate 10

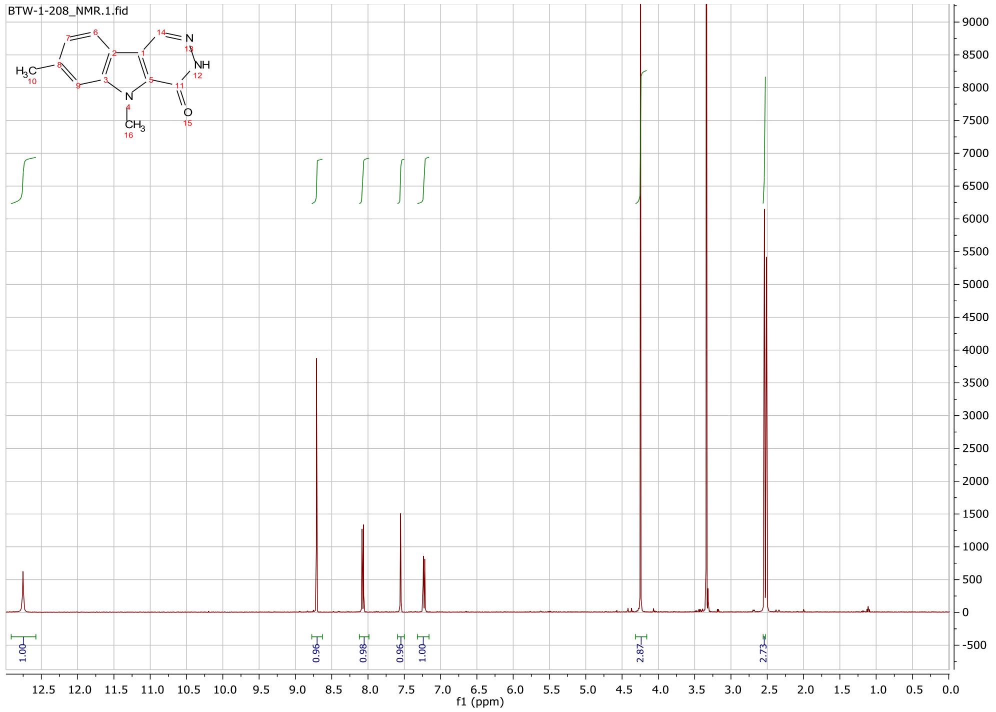

Compound 1

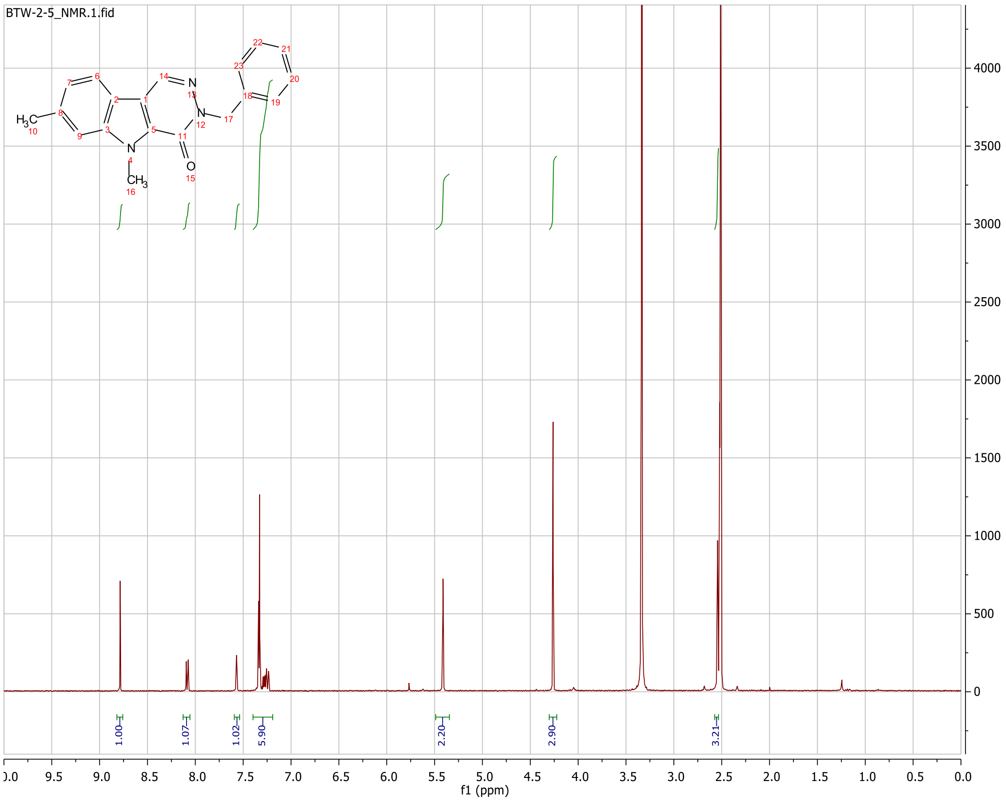

Compound 5

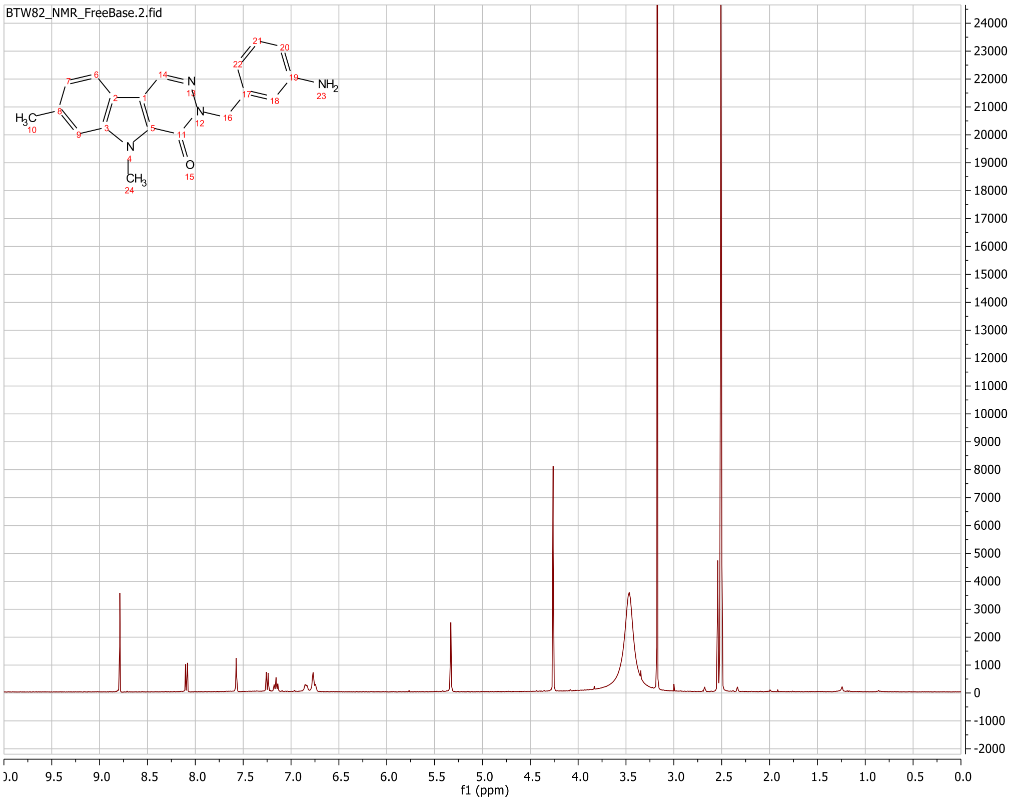

Intermediate 9i

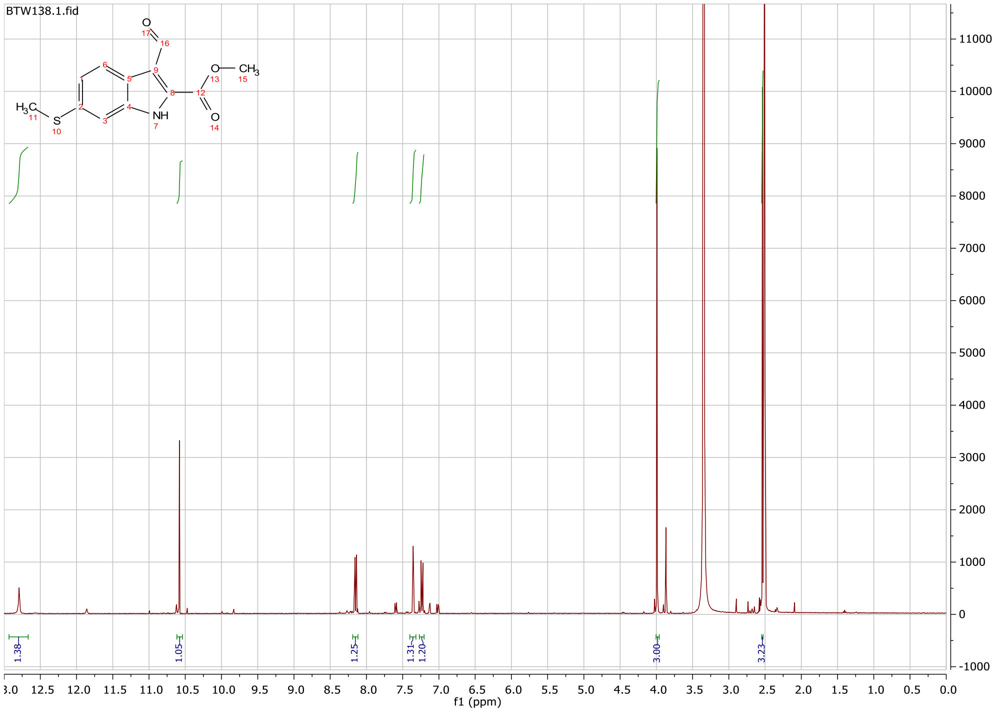

Intermediate 9

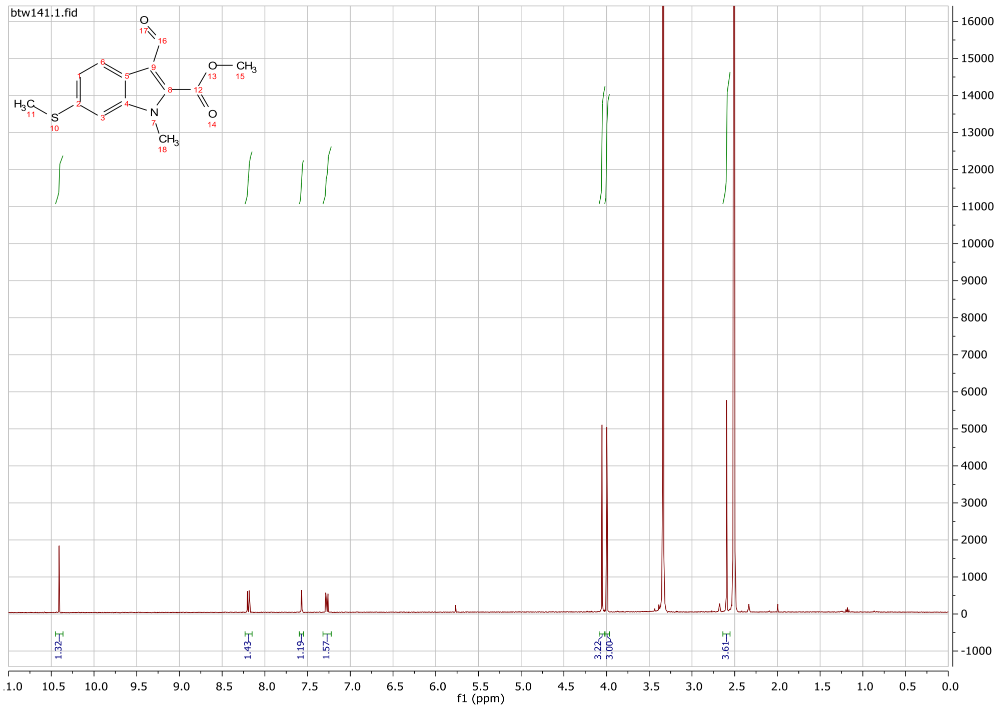

Intermediate 11

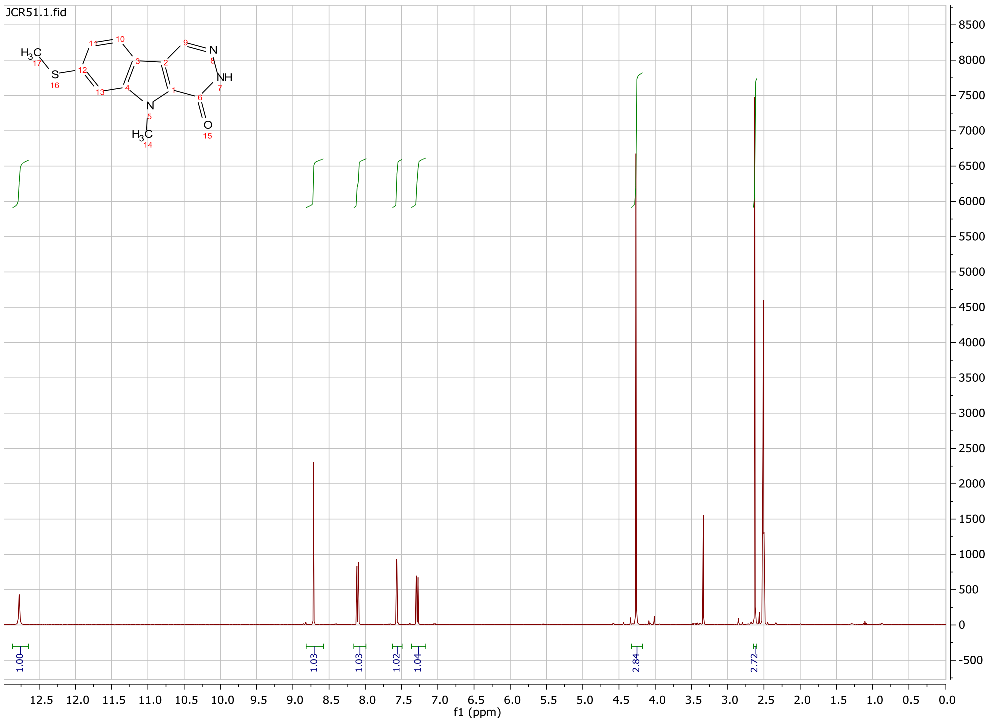

Intermediate 13

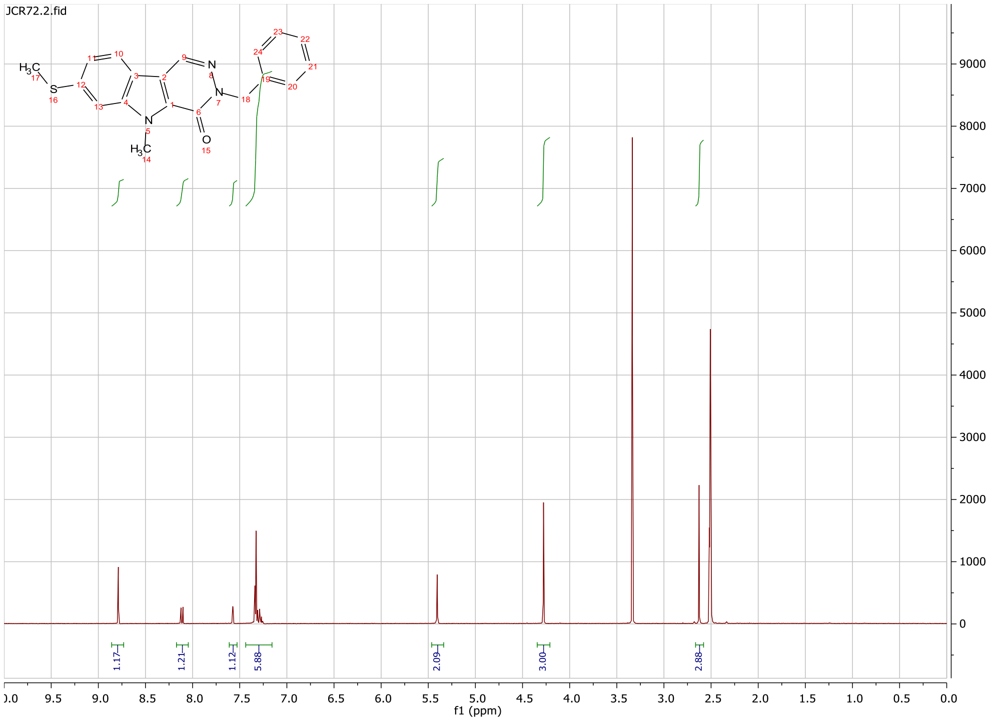

Compound 3

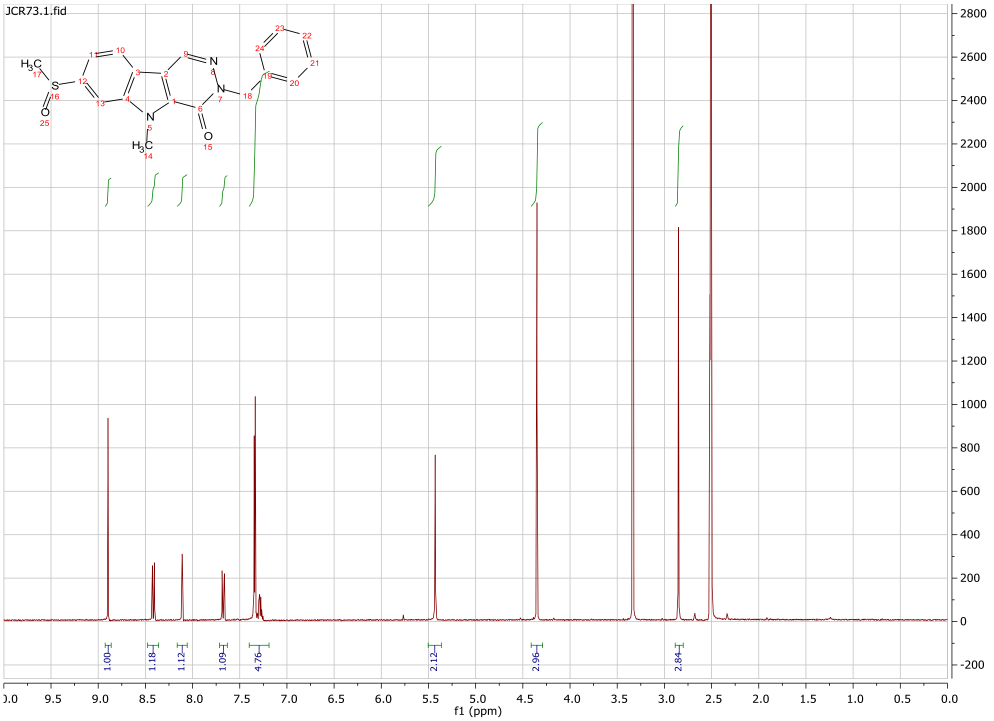

Intermediate 14

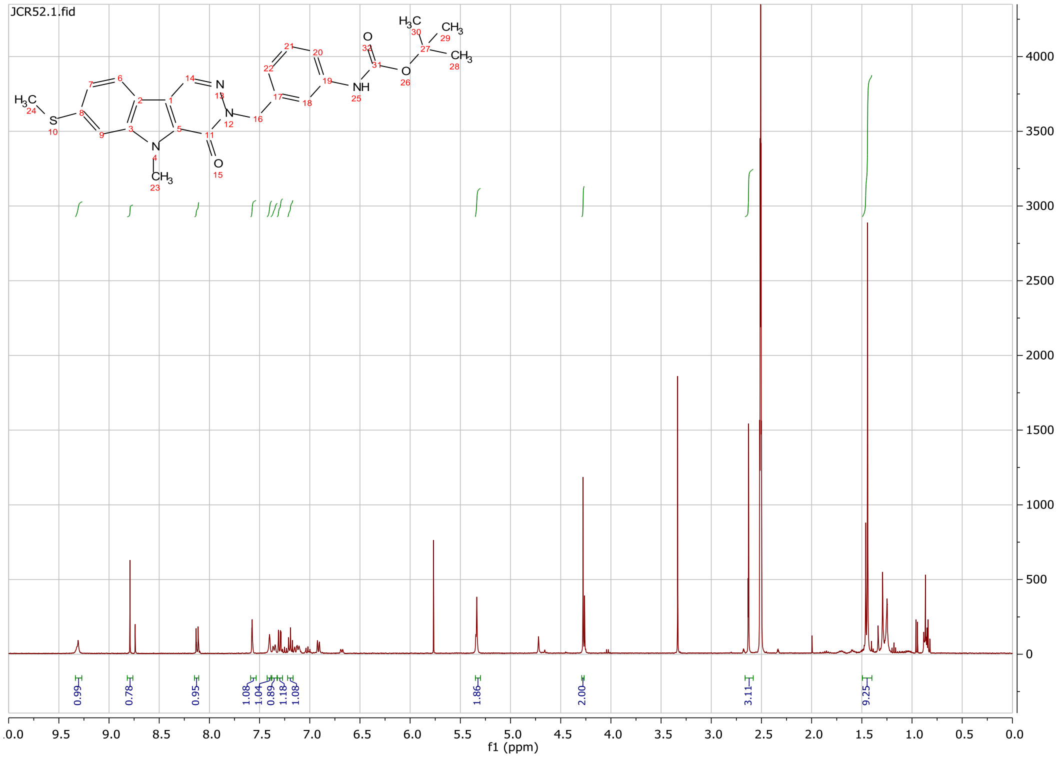

Compound 4

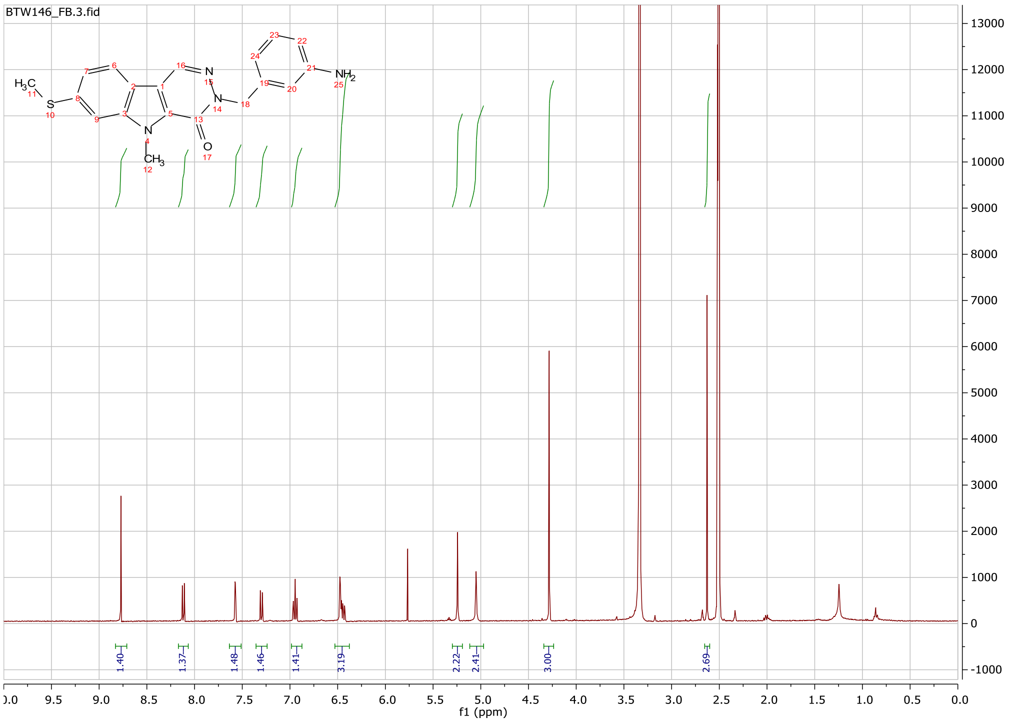

Compound 2

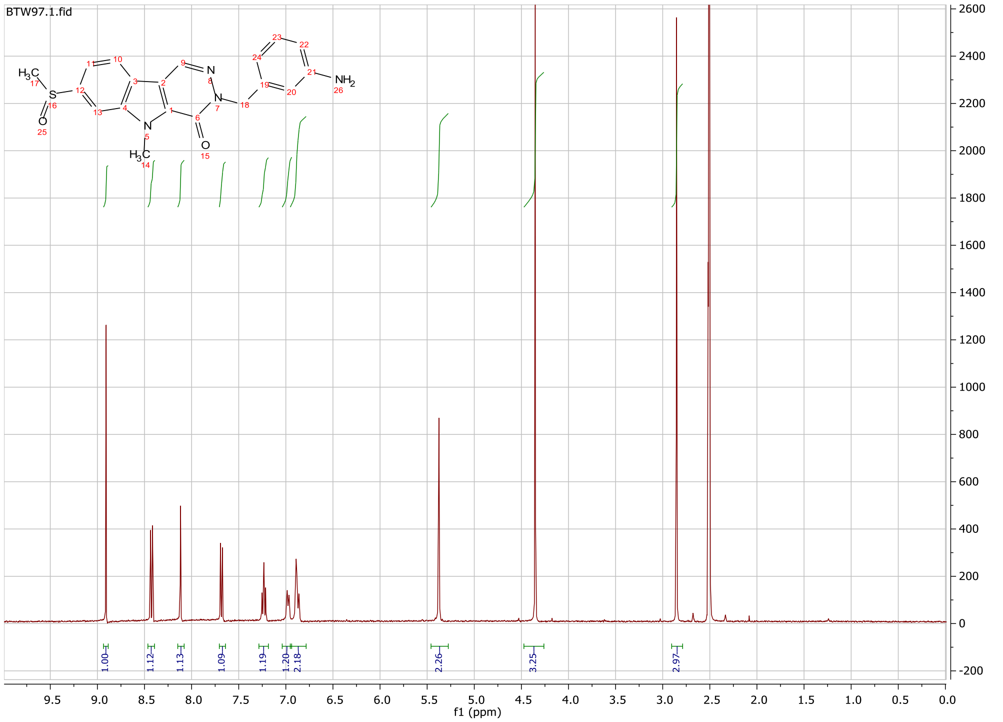

Intermediate 23

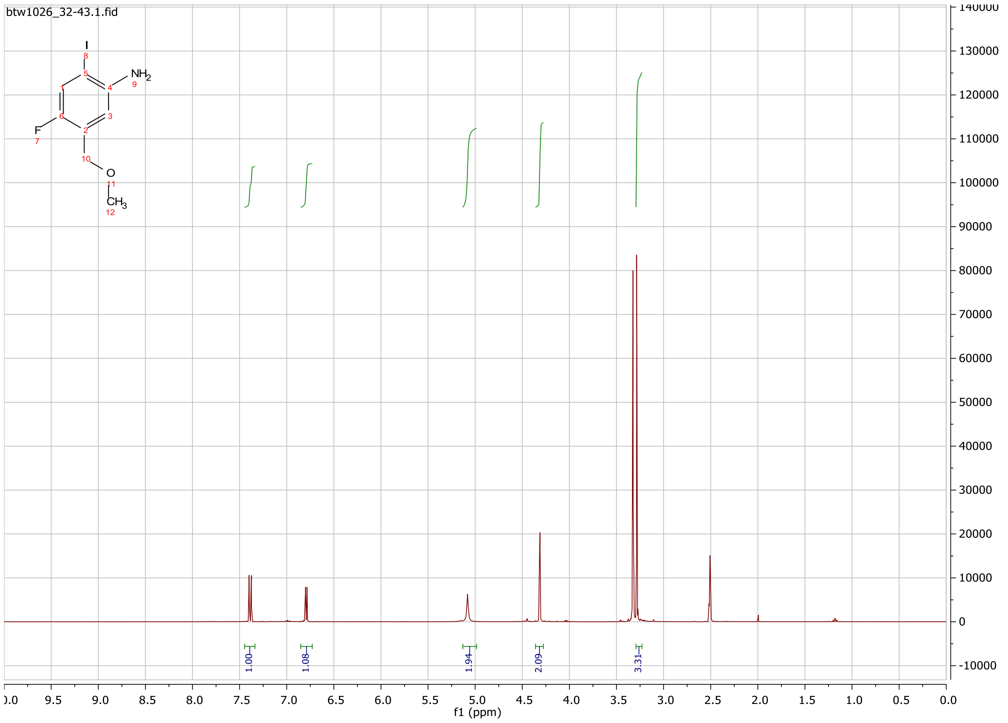

Intermediate 24

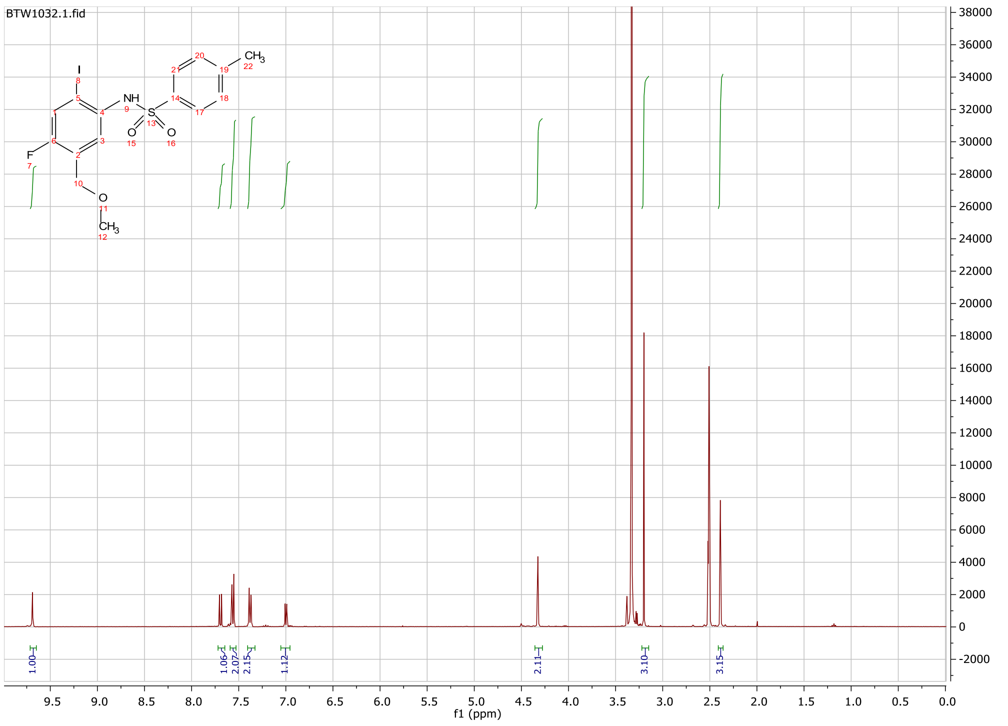

Intermediate 25

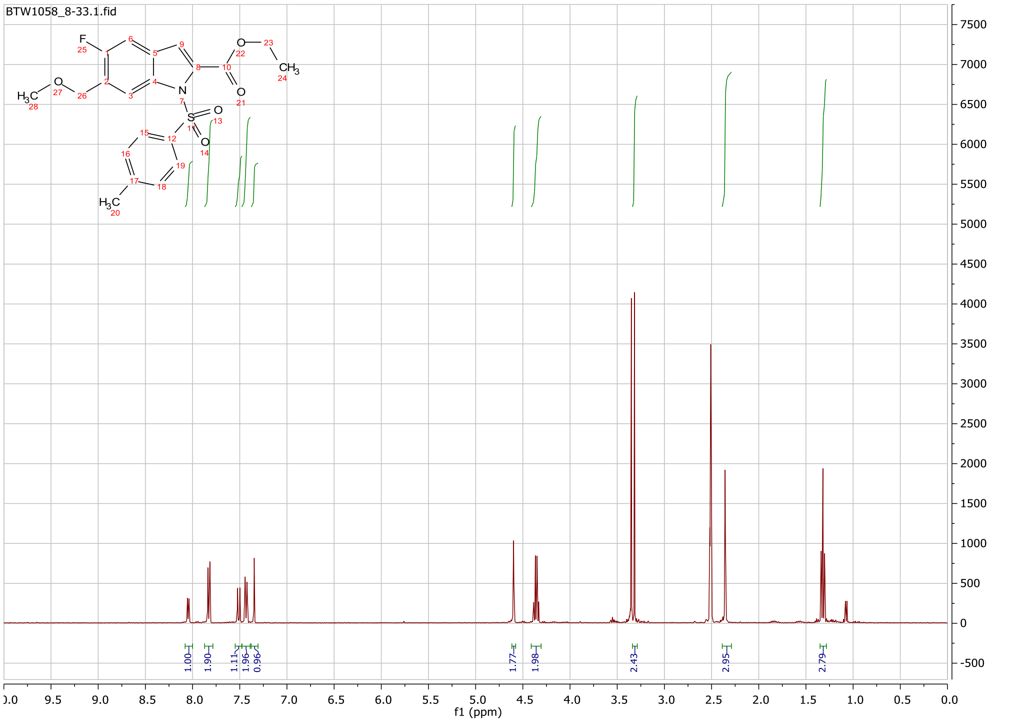

Intermediate 26i

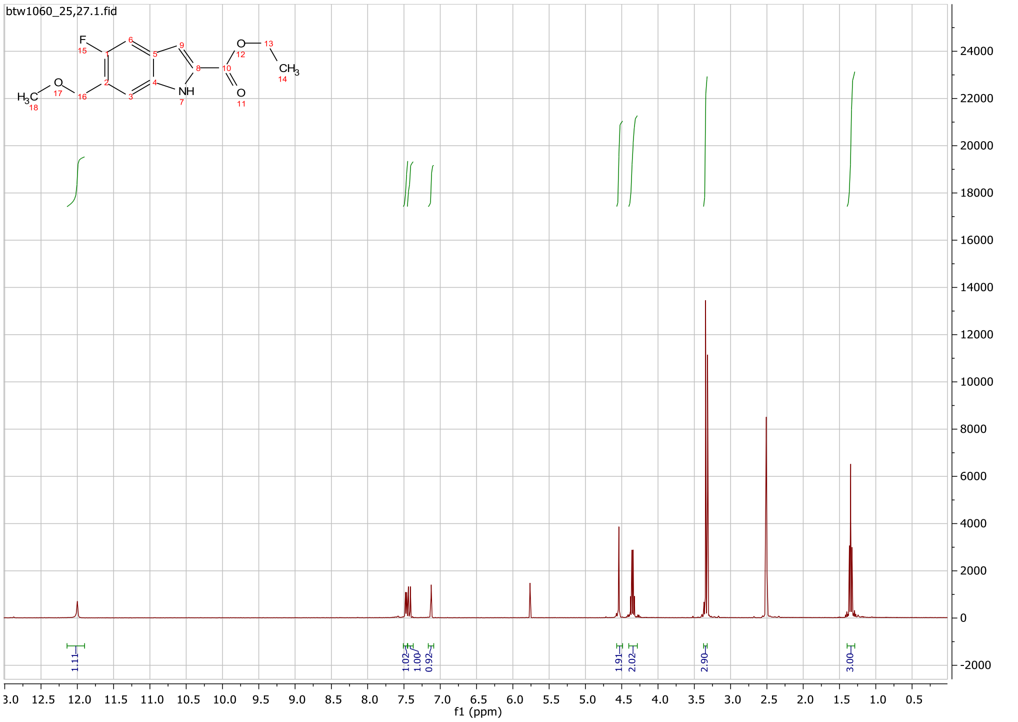

Intermediate 26

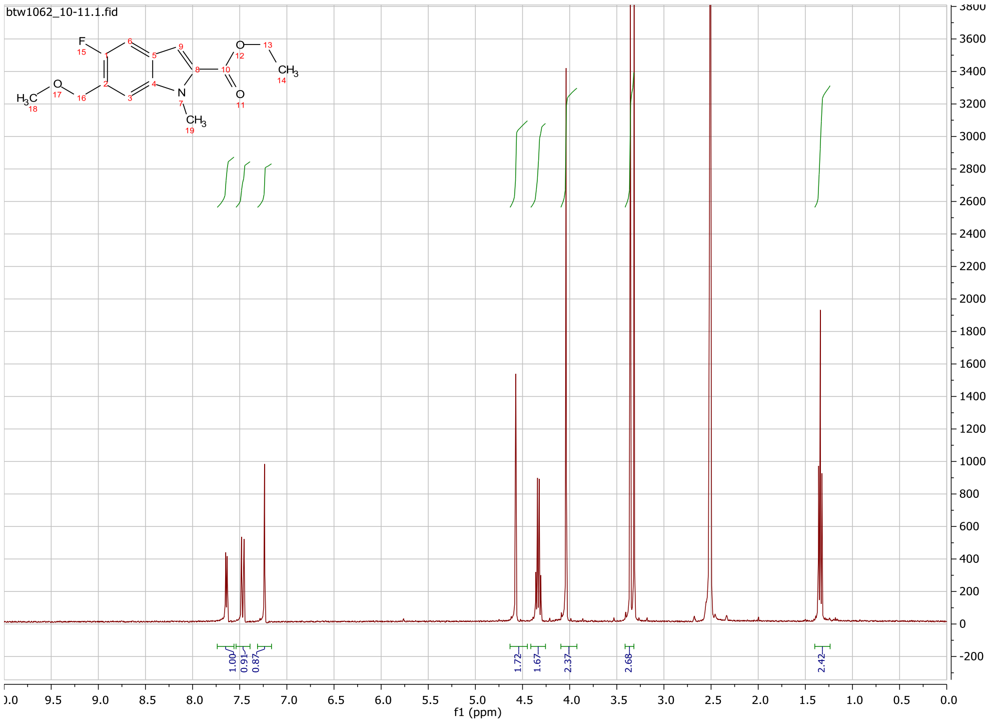

Intermediate 27i

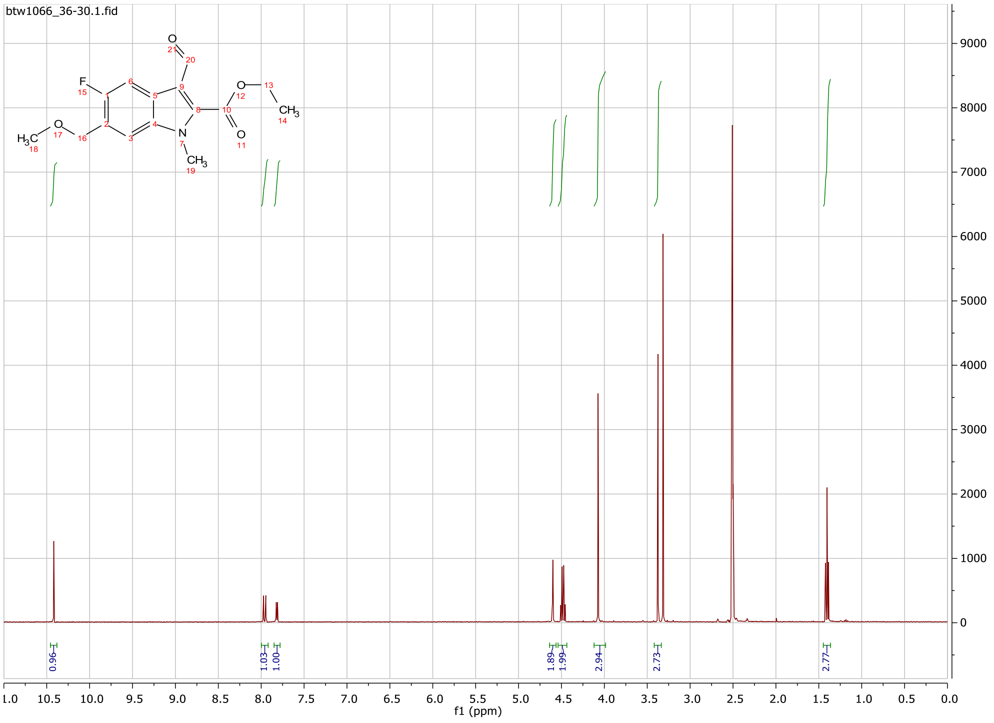

Intermediate 27

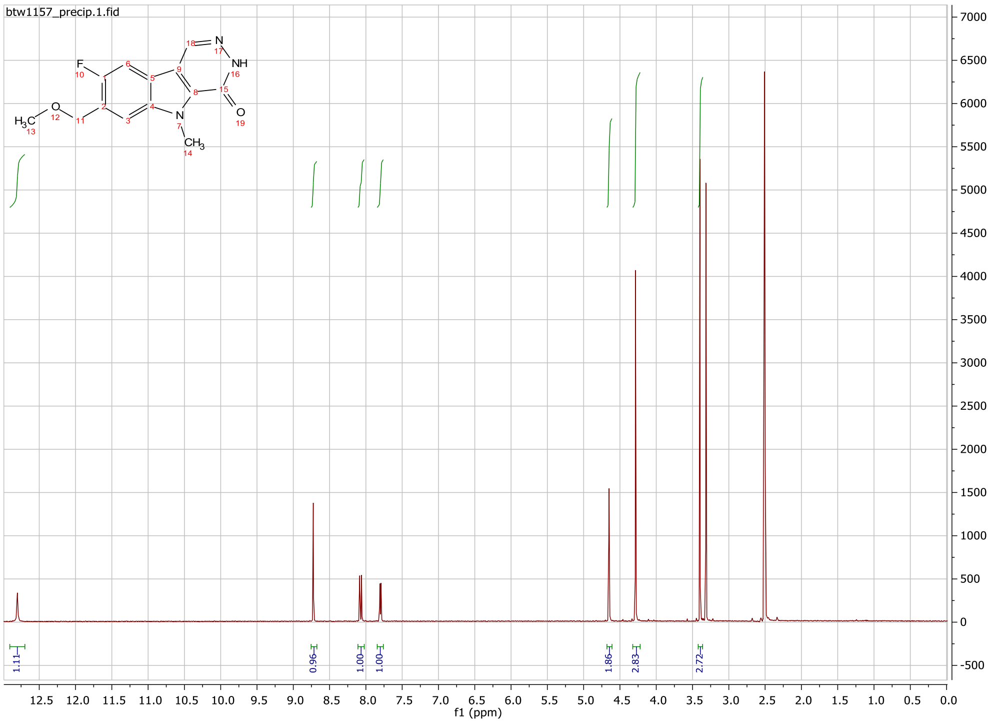

Intermediate 29

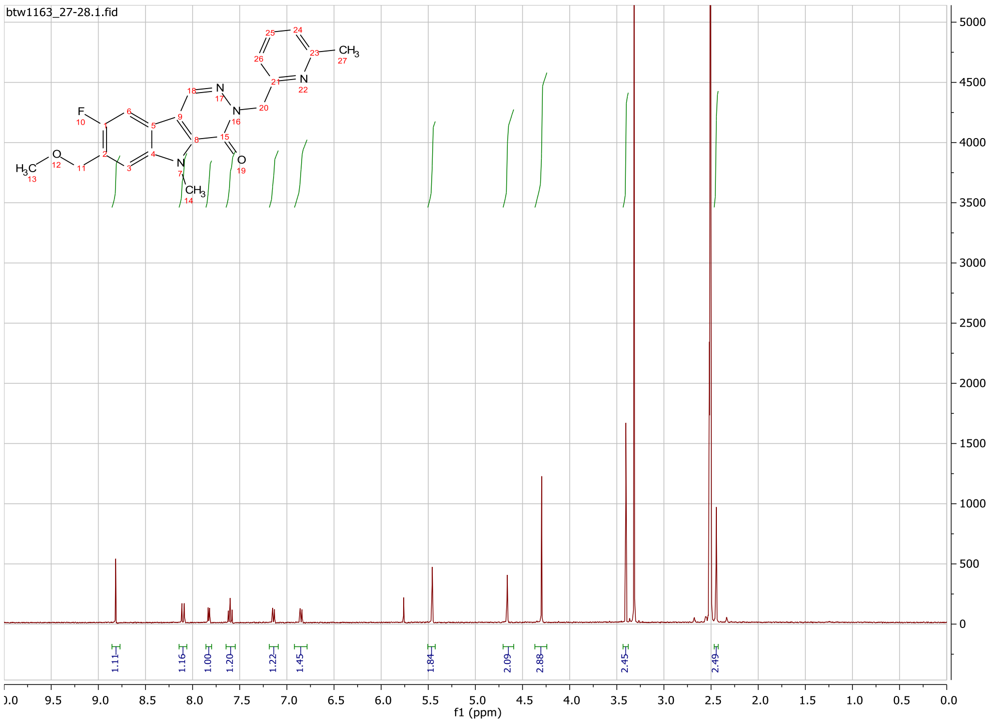

Compound 16

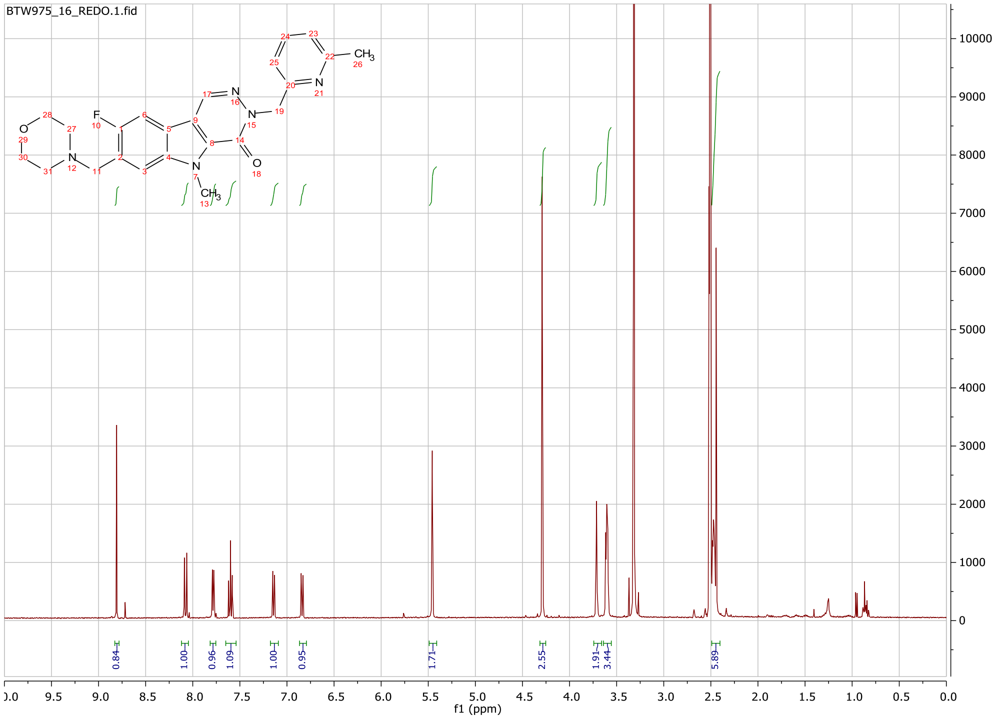

Compound 17

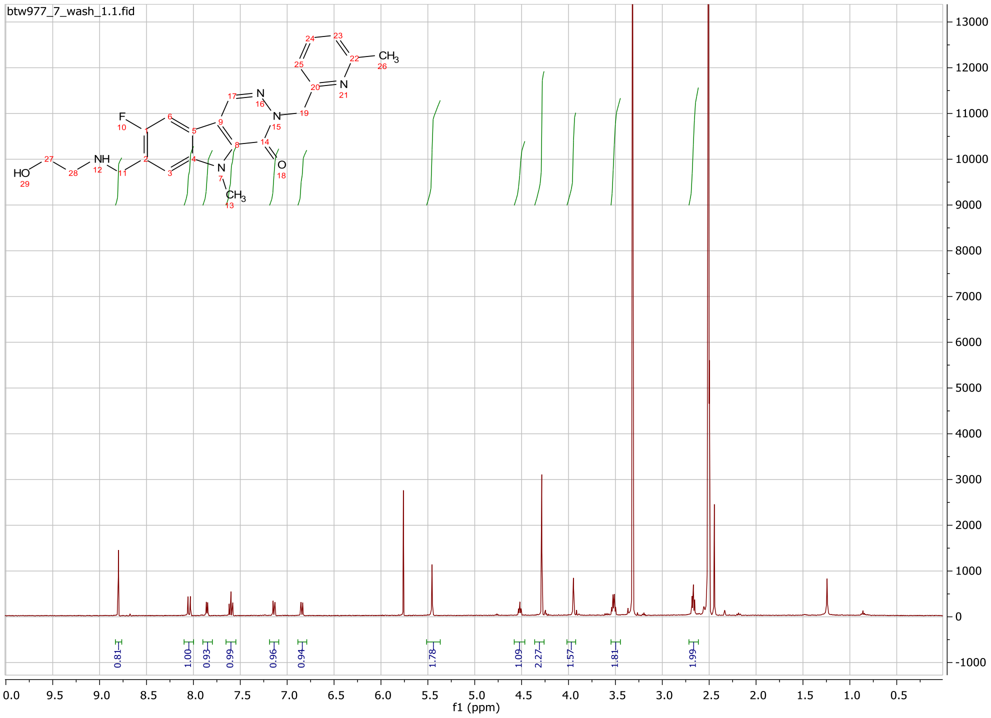

Compound 18

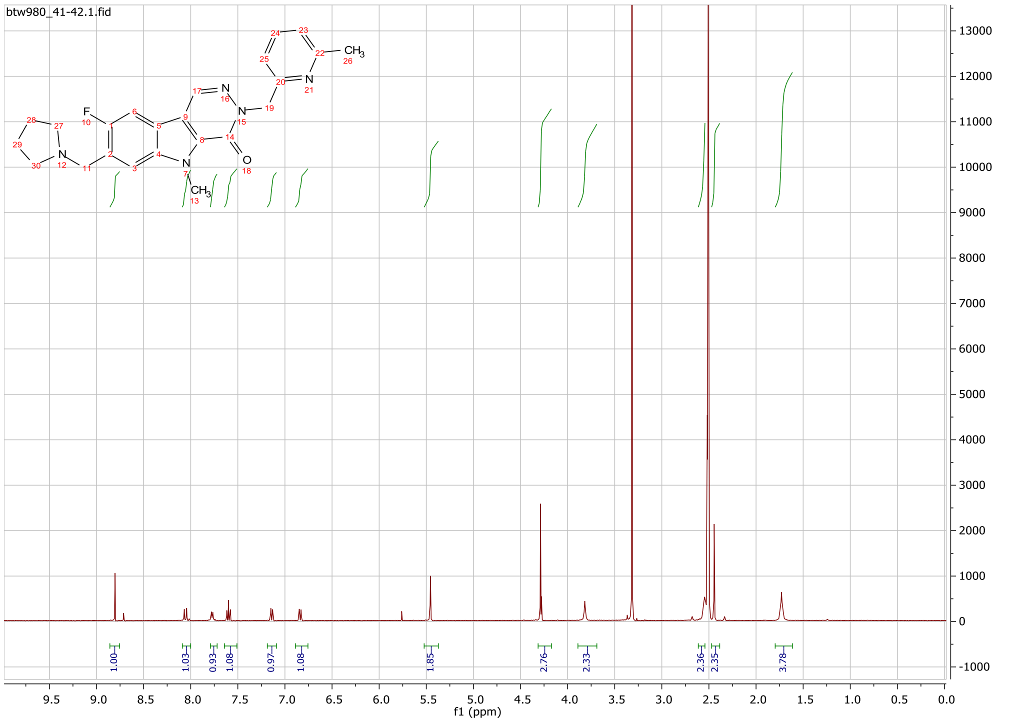

Compound 19

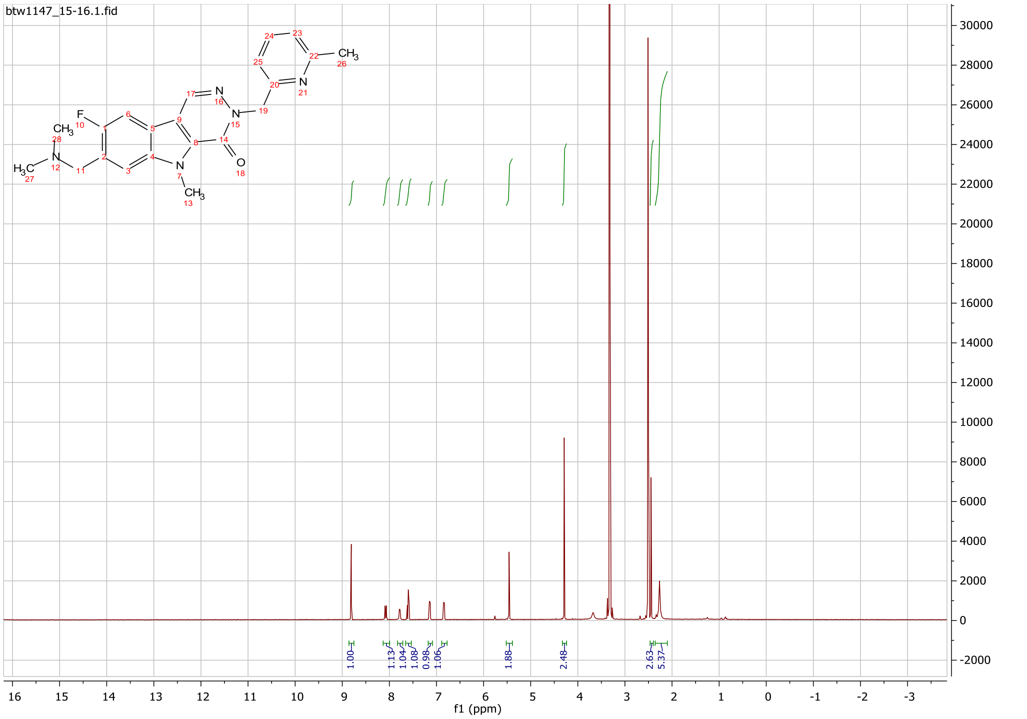

Intermediate 32

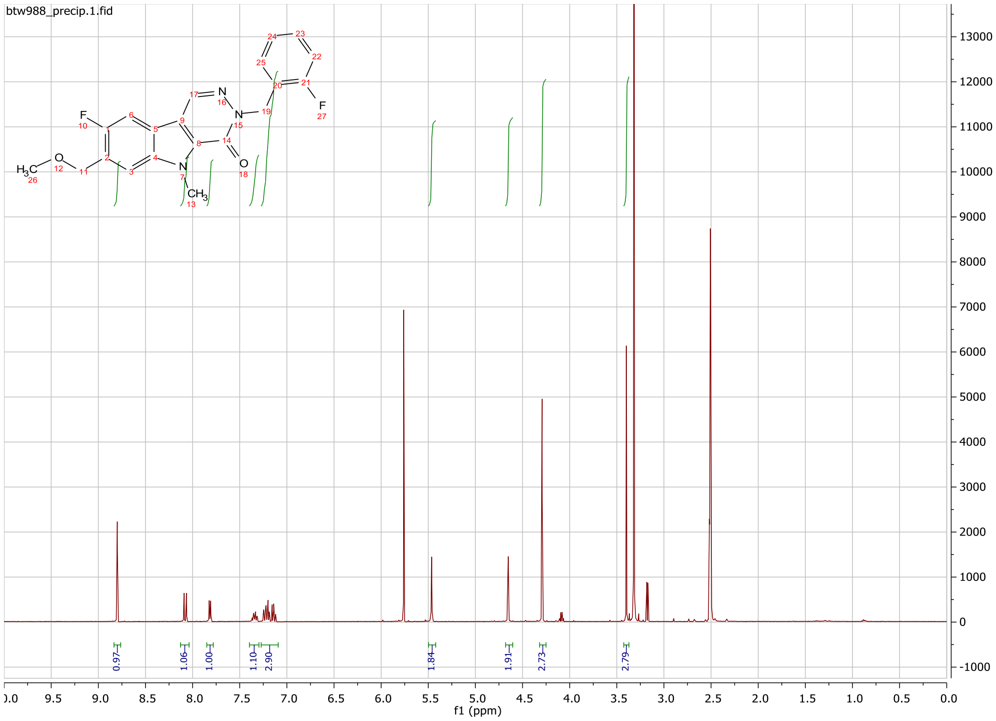

Compound 20

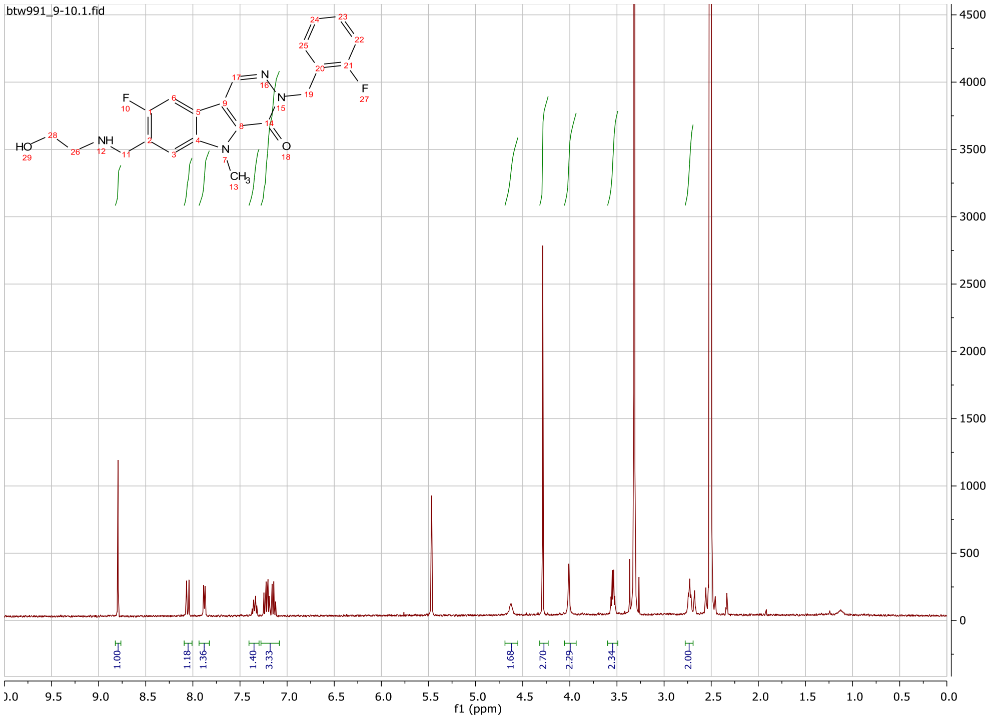

Intermediate 34

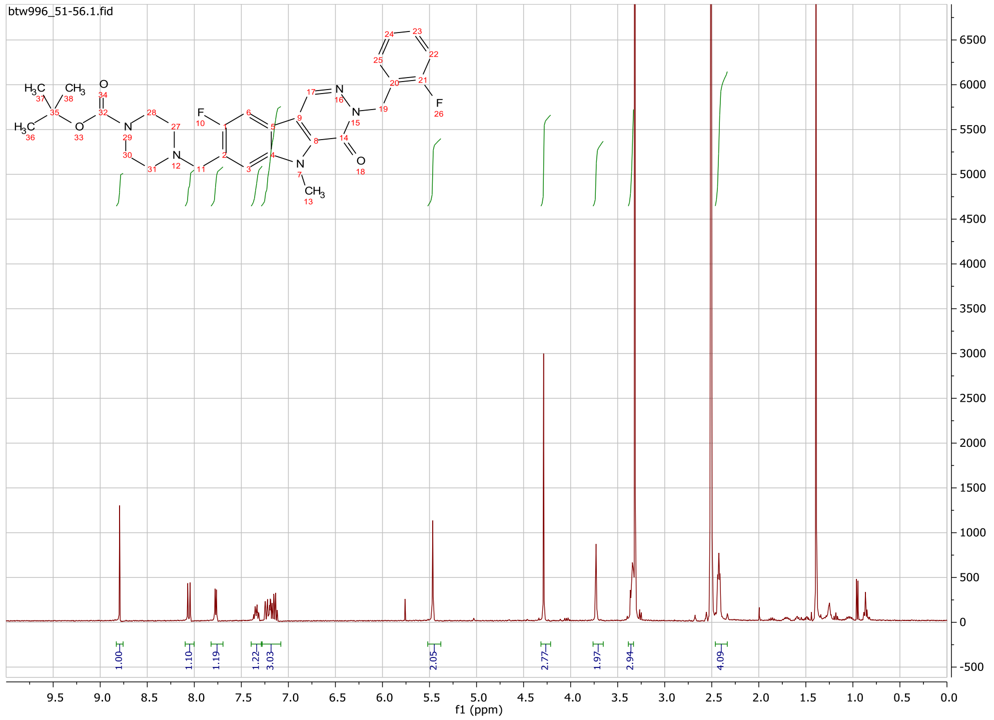

Compound 21

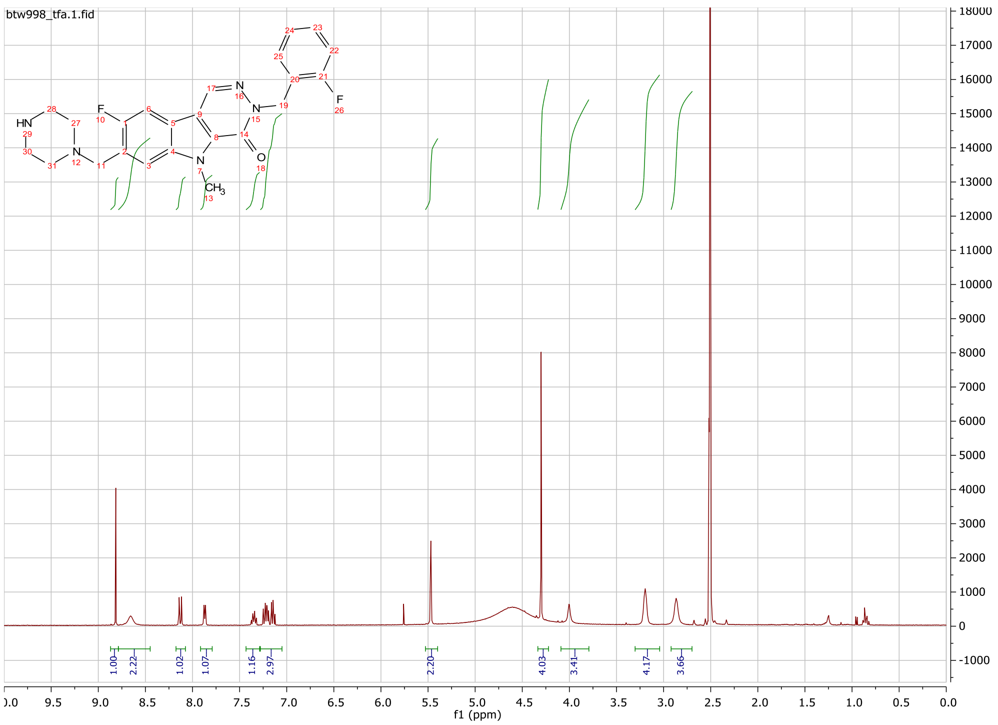

Supplement: Supplementary file 1 [file pharmaceuticals-16-00705-s001.zip › pharmaceuticals-2372405-supplementary.pdf]
